# Defect-engineering of liquid-phase exfoliated 2D semiconductors: stepwise covalent growth of electronic lateral hetero-networks†

**DOI:** 10.1039/d4mh00882k

**Published:** 2024-09-06

**Authors:** Antonio Gaetano Ricciardulli, Christopher E. Petoukhoff, Anna Zhuravlova, Adam G. Kelly, Chun Ma, Frédéric Laquai, Jonathan N. Coleman, Paolo Samorì

**Affiliations:** a Université de Strasbourg, CNRS, ISIS UMR 7006 8 allée Gaspard Monge 67000 Strasbourg France samori@unistra.fr; b King Abdullah University of Science and Technology (KAUST), Physical Science and Engineering Division (PSE), KAUST Solar Center (KSC) Thuwal 23955-6900 Kingdom of Saudi Arabia; c School of Physics, Centre for Research on Adaptive Nanostructures and Nanodevices (CRANN) and Advanced Materials and Bioengineering Research (AMBER), Trinity College Dublin Dublin 2 Ireland

## Abstract

Two-dimensional (2D) in-plane heterostructures display exceptional optical and electrical properties well beyond those of their pristine components. However, they are usually produced by tedious and energy-intensive bottom-up growth approaches, not compatible with scalable solution-processing technologies. Here, we report a new stepwise microfluidic approach, based on defect engineering of liquid-phase exfoliated transition metal dichalcogenides (TMDs), to synthesize 2D hetero-networks. The healing of sulfur vacancies in MoS_2_ and WS_2_ is exploited to controllably bridge adjacent nanosheets of different chemical nature with dithiolated conjugated molecular linkers, yielding solution-processed nm-scale thick networks with enhanced percolation pathways for charge transport. In-plane growth and molecular-driven assembly synergistically lead to molecularly engineered heterojunctions suppressing the formation of tightly bound interlayer excitons that are typical of conventional TMD blends, promoting faster charge separation. Our strategy offers an unprecedented route to chemically assemble solution-processed heterostructures with functional complexity that can be further enhanced by exploiting stimuli-responsive molecular linkers.

## Abstract 2

Two-dimensional (2D) in-plane covalent hetero-networks display exceptional optical and electrical properties well beyond those of their pristine components.

## Graphical abstract

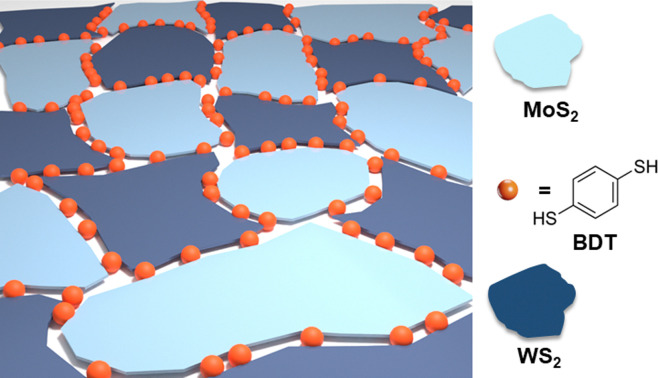

**New concepts**

In this work, we have devised a solution-processed strategy based on defect-engineering to control the in-plane growth of thin hetero-networks from inks of different two-dimensional (2D) materials. To enable the formation of lateral 2D hetero-networks, π-conjugated dithiolated systems have been exploited as molecular linkers to yield MoS_2_–WS_2_ lateral heterojunctions through the healing of sulfur vacancies, mainly located at the edges of the nanosheets. The selective assembly of adjacent MoS_2_ and WS_2_ building units is guaranteed by the design of a cyclic and stepwise microfluidic process based on the sequential deposition of MoS_2_, π-conjugated dithiolated molecules and WS_2_. The synthesized covalent lateral 2D hetero-network displays distinct photophysical properties beyond those of pristine MoS_2_–WS_2_ networks, such as faster charge separation, as well as superior electrical properties than MoS_2_ and WS_2_ covalent homo-networks. Hitherto, the lack of componential selectivity and control over the edge-to-edge assembly of different 2D materials in conventional deposition techniques hindered the progress and exploration of solution-processed heterostructures. Our modular strategy paves the way toward the assembly of endless low-dimensional complex systems endowed with both tailored and uncharted characteristics.

## Introduction

The emergence of two-dimensional (2D) materials, with their thickness down to the atomic scale, has provided access to radically new physical and chemical features.^1–3^ To go beyond the reach of the existing materials and unlock unprecedented paradigms in physics by pursuing functional diversification, 2D materials have been used as atomically thin building blocks to form heterojunctions endowed with tailored functionalities, either in an out-of-plane or in-plane fashion.^4–6^ The latter geometry enables more precise tuning of the heterostructure properties, as the lateral assembly occurs through the edges of the building units, which are more spatially separated.^7^ For instance, p–n nodes can be formed at the atomic scale to shrink further optoelectronic devices, beyond the current limits of Moore's law.^8^ Noteworthily, interfacing diverse transition metal dichalcogenides (TMDs) to form heterostructures has recently attracted much attention as it could either combine the best characteristics of their components^9^ or lead to enticing new physics,^10,11^ envisioning the development of innovative multifunctional (opto)electronic devices based on materials by design.^12,13^ Research on these heterostructures generally relies on physical vapor transport, chemical vapor deposition (CVD), and epitaxial growth techniques, as they offer atomic control over the assembly. Alternatively, solution processing holds promise for the scalable production of large-area architectures for printed technologies. In this context, films made by distinct 2D materials can perhaps be best defined as hetero-networks because their morphology and orientation significantly differ from conventional 2D heterostructures. However, both terms are used throughout the text.

Liquid-phase exfoliation (LPE) features high-yield, cost-effectiveness and scalability, and it outputs nanosheets dispersed in solvents of choice, being the ideal synthetic platform for large-area (opto)electronic devices compatible with printed technologies.^14–16^ Nevertheless, the use of LPE flakes as scaffolds for 2D lateral heterostructures is inhibited by the complete lack of control in terms of componential selectivity and preferential edge-to-edge assembly of different species arising from state-of-the art fabrication strategies, such as spin-coating, spray-coating, drop-casting, *etc.*^17^ Indeed, these techniques yield exclusively thick films of overlapped nanosheets. To eliminate this grand challenge, among the different structural defects typically existing in LPE 2D semiconducting nanostructures, chalcogen vacancies located at the edges of TMD nanosheets, which are the most abundant anomalies and conventionally considered as bottlenecks for charge transport,^18–20^ can be exploited as reactive sites to covalently bridge adjacent flakes to form ultrathin and continuous films through a novel supramolecular strategy.

In this work, to engineer a uniform and large-area TMD-based hetero-network displaying properties well beyond those of the individual components, dithiolated molecules are employed to covalently connect neighboring molybdenum and tungsten disulfide nanosheets (MoS_2_ and WS_2_, respectively) *via* microfluidic flow, which regulates the diffusion of molecules in a controlled manner.^21^ To ensure selective bridging, cyclic and sequential deposition of MoS_2_, π-conjugated dithiolated molecules and WS_2_ is exploited. Moreover, the use of π-conjugated molecular bidentate systems to heal sulfur vacancies in TMDs was demonstrated to be a viable strategy to generate percolation pathways, enabling efficient charge transfer among nanosheets of the same chemical nature, and thus boosting the inter-flake electronic connectivity across the interconnected network.^18^ It is worth noting that, compared to previous studies based on typical deposition methods such as drop-casting or spray coating,^18,22^ the use of a stepwise approach grants access to a larger number of sulfur vacancies in TMDs as the use of low-concentrated dispersions enables flakes to be more spatially separated, resulting in the maximization of defects healing by thiolated molecules. The synergy of multiscale microscopy and both steady-state and time-resolved spectroscopy analyses confirm that the use of such molecular bridges is critical to both control the synthesis of MoS_2_–WS_2_ lateral heterojunctions by exploiting sulfur vacancies as anchoring sites and enhance the overall electrical performance. Indeed, as revealed by photophysics investigations, faster charge separation is observed in such lateral hetero-networks compared to pristine MoS_2_–WS_2_ blends, suggesting new physical characteristics that hold potential in printed (opto)electronics. Moreover, our microfluidic approach grants access to percolation of charges at reduced thicknesses (single flake-thick). Indeed, field-effect transistors (FETs) based on solution-processed hybrid edge-bridged MoS_2_–WS_2_ heterostructures exhibit charge carrier mobilities matching state-of-the-art films made of LPE TMDs, which are typically measured in films displaying orders of magnitude higher thicknesses. The modular strategy devised in this work can be readily extended to the whole library of solution-processed low-dimensional nanomaterials, including 0D, 1D and 2D structures, and *ad hoc* molecular linkers with tailored multifunctional moieties, and thus serve as a powerful toolkit to assemble endless complex heterostructures with on-demand functionalities through geometrically controlled growth.

## Results and discussion

### Synthesis of solution-processed 2D hetero-networks through defects

**Fig. 1:**
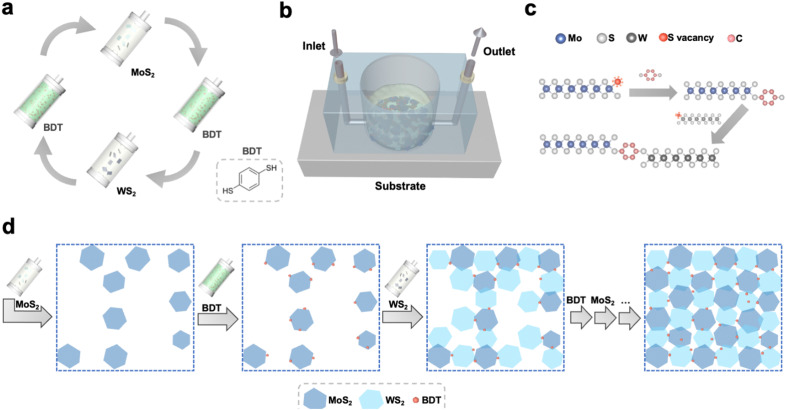
Covalent hetero-network formation strategy. (a) Alternating steps of the microfluidic cycle. The process is repeated until the formation of an interconnected in-plane network. To minimize aggregation, IPA is injected between each step. (b) Schematic representation of the microfluidic setup. (c) Illustration of the healing of edge sulfur vacancies, exploited to bridge adjacent TMD flakes by BDT. (d) Schematic top-view of the stepwise growth of the lateral hetero-network, made of MoS_2_–BDT–WS_2_ core units. The last arrow illustrates that further alternating steps, following the cycle displayed in Fig. 1a, are repeated to yield the film.

**Fig. 2:**
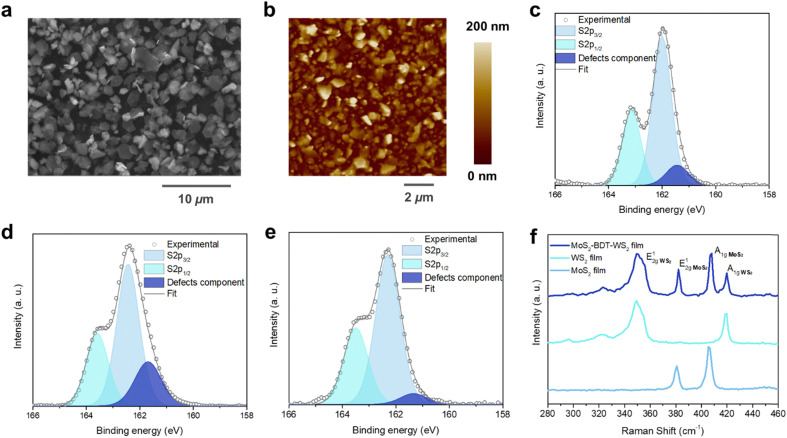
Morphological and steady-state spectroscopical investigation. (a) SEM image of a film based on MoS_2_–WS_2_ heterostructure after the optimized process. (b) AFM topographical images on randomly selected areas of the heterostructure. High-resolution S 2p XPS spectrum of (c) pristine MoS_2_, (d) pristine WS_2_ and (e) hybrid MoS_2_–WS_2_ lateral hetero-network. (f) Raman spectra comparison of pristine MoS_2_ and WS_2_ with hybrid MoS_2_–WS_2_ lateral heterostructure.

The processing steps to grow the 2D networks based on defect-engineered LPE MoS_2_ and WS_2_ are illustrated in Fig. 1. To avoid the randomness of material deposition that occurs with conventional fabrication methods (*i.e.* drop-casting, spin-coating, spray-coating, *etc.* – ESI,† Fig. S1) and maximize the amount of MoS_2_–WS_2_ heterojunctions, the synthesis of the heterostructure was carried out under laminar flow assisted by a sequential microfluidic process, using tailored conditions (Fig. S2–S4, ESI†). To demonstrate the validity of the sequential approach to grow heterostructures from solution-processed materials, a basic microfluidic system was set up. First, LPE MoS_2_ ink was flowing on top of a Si/SiO_2_ substrate, whose wettability was previously tuned *via* the chemisorption of a 3-(aminopropyl)triethoxysilane (APTES) monolayer to facilitate the adhesion of MoS_2_. Subsequently, 1,4-benzenedithiol (BDT) was used to control the *in situ* formation of the heterojunctions. The use of a conformationally rigid, bidentate molecule is pivotal to assemble the heterostructure. On the one hand, chalcogen vacancies exposed on the deposited MoS_2_ nanosheets, primarily located at the edges of 2D TMDs, act as anchoring sites for one of the two thiol-terminals of BDT. On the other hand, the second unreacted thiol end-group of the anchored BDT can be used to link covalently further nanosheets in the subsequent step. Thus, LPE WS_2_ ink was introduced in the microfluidic chamber to build the edge-to-edge MoS_2_–WS_2_ heterojunctions *via* BDT bridging. Finally, BDT was further employed to covalently functionalize the exposed sulfur vacancies at the edges of deposited WS_2_. The alternating steps result in a microfluidic cycle that is repeated multiple times to yield an interconnected, uniform and thin film with a coverage of ∼70% (Fig. S5, ESI†). This feasible and scalable solution-processed growth represents the first example of liquid-phase, structurally controlled, formation of an in-plane heterostructure of 2D materials, exploiting structural defects at the edges of TMDs to drive the preferentially in-plane heterojunction formation by covalently bridging the flakes of the network with dithiolated molecules. While the intrinsic conformational rigidity of the BDT molecules hinders backfolding and linkage of the two thiol end-groups to the same nanosheet, despite the control over the multiple steps of this solution-processed approach, unintentional vertical overlaps of flakes yielding homojunction formations may still occur. The morphology of the hybrid MoS_2_–WS_2_ heterostructure was investigated by scanning electron microscopy (SEM) and atomic force microscopy (AFM). To design the optimal procedure for the formation of a smooth solution-processed film, SEM analysis was used to track the nanosheets arrangement after each LPE TMD deposition step (Fig. S6, ESI†). It should be noted that the interconnected film coverage of ∼70% represents the best trade-off in terms of both heterojunction formation and thickness (Fig. 2a) as further addition of either MoS_2_ or WS_2_ nanosheets would result in a physisorption-driven 3D growth through the overlap of flakes (Fig. S7, ESI†), since the sulfur vacancies of deposited nanosheets become less accessible as a result of the dense packing of the coating. The smoothness of the thin solution-processed lateral hetero-network is further verified by AFM imaging (Fig. 2b).

#### Molecular and defect engineered MoS_2_–WS_2_ hetero-networks

The effective *in situ* functionalization of both LPE MoS_2_ and WS_2_ with BDT was further assessed by X-ray photoelectron spectroscopy (XPS) and Raman spectroscopy measured in various positions over multiple samples. XPS analysis was carried out on BDT-bridged MoS_2_–WS_2_ heterostructure as well as on pristine LPE MoS_2_ and WS_2_ flakes. Notably, high-resolution S 2p spectrum of TMDs containing sulfur as chalcogen unveils the defectivity of the system, thus providing a tool for probing the healing of sulfur vacancies upon selective chemical functionalization.^23,24^Fig. 2c and d shows the core level S 2p spectra of pristine LPE MoS_2_ and WS_2_ films, displaying the main two S 2p_3/2_ and S 2p_1/2_ components of MoS_2_ (∼162.0 eV and ∼163.2 eV) and WS_2_ (∼162.4 eV and ∼163.6 eV), respectively. A third component for both MoS_2_ and WS_2_ at ∼161.4 eV and ∼161.7 eV, respectively, is further deconvoluted and ascribed to the defects of the crystal structure, such as the vacancy of neighboring sulfur atoms.^25,26^ The significant reduction of the relative area of the defects-related component in the BDT-functionalized lateral heterostructure (Fig. 2e), from 7.2 ± 1.4% and 25.3 ± 2.1% for MoS_2_ and WS_2_, respectively, to 2.8 ± 1.6%, clearly indicates the decrease of sulfur vacancies, suggesting the efficient healing through BDT covalent functionalization. In contrast, the defective component in MoS_2_–WS_2_ bi-component films without BDT treatment, displays a relative area of 9.0 ± 0.2% (Fig. S8, ESI†). Further evidence is provided by comparing the substoichiometric Mo 3d and W 4f spectra of BDT-functionalized MoS_2_–WS_2_ heterostructure and the pristine LPE MoS_2_–WS_2_ blend (Fig. S9 and S10, ESI†). The Raman spectra taken from selected locations of the lateral heterostructures are composed of an overlay of the resonance modes of both MoS_2_ and WS_2_ domains (Fig. 2f). Noteworthy, the characteristic Raman features of MoS_2_ and WS_2_ are preserved, suggesting that the interaction with BDT does not induce any apparent change to the intrinsic structural characteristics of the materials. Furthermore, the Raman mapping on the film reveals an equal distribution of both 2D components, indicating an intimate mixing of MoS_2_ and WS_2_ junctions (Fig. S11 and S12, ESI†), thus the absence of a phase segregation between the different components that would take place through a simple mixing. However, the defective presence of homojunctions cannot be fully excluded. In addition to XPS findings, the healing of sulfur vacancies by thiolated molecules is also endorsed by Raman analysis. The full width at half maximum (FWHM) of the E^1^_2g_ and A_1g_ peaks of both MoS_2_ and WS_2_ witnesses a considerable reduction (as high 32.9% and 20.9% for the E^1^_2g_ and A_1g_ modes of MoS_2_, 6.7% and 10.1% for the E^1^_2g_ and A_1g_ modes of WS_2_, respectively. Table S1, ESI†) compared to their analogues in pristine LPE materials (Fig. 2f).^27^ Moreover, the spectra of the heterostructure display a considerable blue shift of the E^1^_2g_ and A_1g_ Raman features (Fig. S13, ESI†), indicating the suppression of defect-induced modes, and thus being consistent with a reduction of sulfur vacancies upon BDT functionalization.^28,29^ The shift of the characteristic Raman bands observed in this work exceeds those observed in previous studies on defects passivation in TMDs by thiolated species,^18,22^ indicating a considerable increase of sulfur vacancies healing as nanosheets are more accessible by using a simple stepwise microfluidic-assisted strategy compared to typical deposition techniques that yield overlapped and stacked flakes, limiting the access to sulfur vacancies.

#### *In situ* electrochemical impedance spectroscopy

**Fig. 3:**
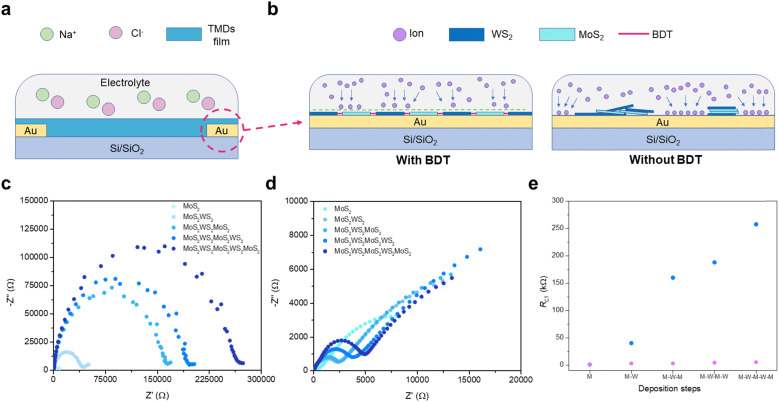
Tracking the growth of heterostructures *via in situ* EIS. (a) Schematic illustration of the electrochemical cell used for EIS. (b) Representation of TMDs network acting as a barrier to ionic conductivity with (left) and without BDT (right). (c) Nyquist plot evolution when BDT is inserted in the microfluidic chamber between each MoS_2_ and WS_2_ step. (d) Nyquist plots after each TMD deposition step for MoS_2_–WS_2_ blends in absence of BDT. (e) *R*_ct_ comparison as a function of TMD deposition for MoS_2_–WS_2_ films with and without BDT, displayed in blue and purple, respectively.

Electrochemical impedance spectroscopy (EIS) was used to elucidate the synergistic effect of employing dithiolated molecules to propel the heterostructure development through a preferential in-plane growth and promote the creation of percolation pathways, as this technique is extremely sensitive to small variations within an electrochemical system. As illustrated in Fig. 3a, a tailored 2-electrode electrochemical cell was designed for conducting *in situ* EIS. The growing heterostructure onto patterned interdigitated electrodes on APTES-modified Si/SiO_2_ substrates acts as a barrier to the ionic conductivity (Fig. 3b), which is triggered by the application of a fixed sinusoidal voltage across the system (Fig. S14, ESI†). As the steady growth of the 2D assembly hampers the ionic conductivity between electrodes,^30^*in situ* EIS measurements exhibit a significant increase of impedance after each TMD deposition sequence (Fig. 3c), thus acting as internal gauge providing the real-time tracking of stepwise film formation *via* microfluidic approach. The findings indicate larger planar coverage within the interdigitating electrodes is achieved, as a result of the BDT-linking effect within adjacent flakes, propelling the favoured lateral assembly. To validate the critical role of bidentate molecules to assemble the in-plane heterostructure, EIS was performed after each MoS_2_ and WS_2_ deposition in the absence of BDT as an intermediate process. Transient impedance did not display any significant change upon TMD-loading increase, suggesting partial and uneven coverage of the surface among the interdigitating electrodes (Fig. 3d). A standard Randles equivalent circuit is used to fit the Nyquist plots (model and further discussion in ESI,† Fig. S14) and extract the charge transfer resistance (*R*_ct_),^31^ which is the resistance to the ionic conduction encountered at the interface between electrolyte and electrode. The architecture integrating the MoS_2_–WS_2_ heterostructure mediated by BDT witnesses a large contribution of the *R*_ct_ along with the in-plane growth of the network (Fig. 3e). In contrast, the bare deposition of nanosheets without employing any molecular bridge leads to reduced *R*_ct_ variation, being consistent with irregular and scarce coating of MoS_2_ and WS_2_ flakes. Typical SEM images of the film fabricated by alternating only MoS_2_ and WS_2_ nanosheets provide a distinct footprint of such EIS findings (Fig. S15, ESI†). To exclude the effect of benzene rings in the increase of *R*_ct_ and further validate the in-plane growth of the heterostructure mediated by BDT, EIS has been carried out in films where thiophenols have been introduced to heal individual sulfur vacancies in MoS_2_ and WS_2_ after each deposition step, with the equivalent procedure adopted with BDT. Likewise, for the film formed by alternating MoS_2_ and WS_2_ only, thiophenol-functionalized coatings exhibit a reduced *R*_ct_ comparable to the films without any molecular linker (Fig. S16, ESI†). Indeed, despite the MoS_2_ and WS_2_ functionalization through the vacancies,^18^ thiophenols are not able to bridge adjacent flakes due to the absence of a second thiol like in BDT, inhibiting the formation of a MoS_2_–WS_2_ network.

### Photophysics of MoS_2_–WS_2_ heterostructures

**Fig. 4:**
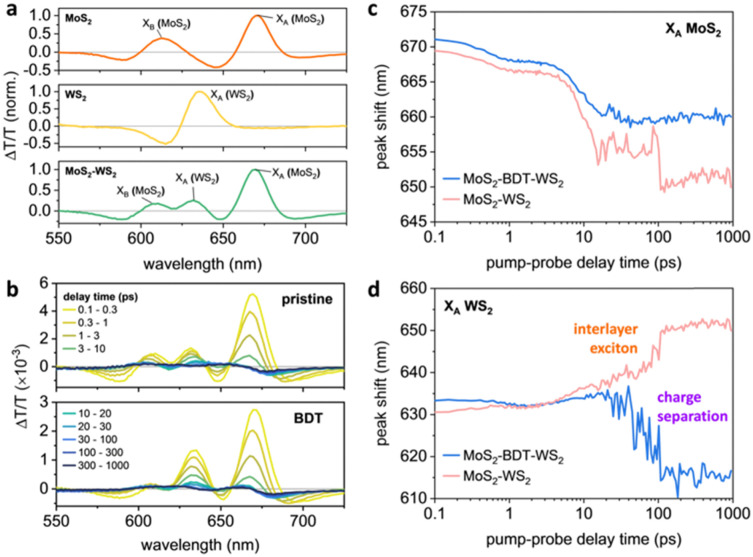
Transient absorption (TA) spectra and peak shifts. (a) TA spectra of pristine MoS_2_, WS_2_, and MoS_2_–WS_2_ heterostructure averaged over pump–probe delay times of 0.1–0.3 ps. The dominant species observed within this spectral range are indicated. (b) TA spectra of MoS_2_–WS_2_ heterostructures, with or without BDT. The spectra are averaged over the pump–probe delay times listed in the legend. (c) and (d) Peak shifts for the reported species in the MoS_2_–WS_2_ heterostructures, with or without BDT, as determined from Gaussian fits to the individual peaks: (c) *X*_A_ of MoS_2_; (d) *X*_A_ of WS_2_.

As BDT enables the sequential linking of adjacent flakes, altering the overall morphology of the microfluidic-assisted grown heterostructure and regulating the distribution of its components, the impact of using such molecular linker on the overall photophysical characteristics of the synthesized MoS_2_–WS_2_ hetero-networks was revealed by transient absorption (TA) pump–probe spectroscopy studies. The TA spectra from pristine MoS_2_ and WS_2_ are dominated by their excitonic resonances: particularly the A-exciton (*X*_A_) and B-exciton (*X*_B_) from MoS_2_, and the *X*_A_ from WS_2_ within the range of 550 nm to 725 nm (Fig. 4a and Fig. S17, S18, ESI†).^32–34^ The spectra have alternating negative and positive differential transmission (Δ*T*/*T*) signals, arising from spectral shifts and broadening of the photoexcited states relative to the ground state due to bandgap renormalization.^32–35^ The TA spectra of the MoS_2_–WS_2_ heterostructures are composed of signals from component materials (Fig. 4a). For the BDT-linked heterostructures, the ratio of *X*_A_ (WS_2_): *X*_A_ (MoS_2_) increased, indicating a higher ratio of WS_2_ was present in the linked heterostructures (Fig. 4b). As pump–probe delay time increased, the peaks from each of the excitons shifted spectrally, with each of the excitons in the pristine materials blue-shifting as their bandgaps returned to their ground-state values (Fig. S18–S20, ESI†). This was similar for MoS_2_'s *X*_A_ signals in the heterostructures (Fig. 4c); however, MoS_2_'s *X*_B_ and WS_2_'s *X*_A_ exhibited different trends (Fig. S19b and Fig. 4d, respectively, ESI†). For the unlinked heterostructure, the WS_2_'s *X*_A_ red-shifted with increasing pump–probe delay times – indicating further narrowing of WS_2_'s bandgap upon formation of a long-lived interlayer exciton between MoS_2_ and WS_2_, which is typical for vertical heterostructures.^36^ This was not the case for the BDT-linked heterostructure: in this case, WS_2_'s *X*_A_ blue-shifted back to its ground state value in ∼100 ps, further suggesting the different architecture through the preferential in-plane growth mediated by BDT. This rapid blue shift of WS_2_'s *X*_A_ indicates the lack of formation of tightly bound interlayer excitons, indicating that the BDT-linked heterostructures promote faster charge separation. This was further evidenced by the faster relaxation dynamics of WS_2_'s *X*_A_ in the BDT-linked heterostructures compared to the unlinked heterostructures (Fig. S21 and Table S2, ESI†), suggesting potential applications in light harvesting systems.

### Electrical characteristics

**Fig. 5:**
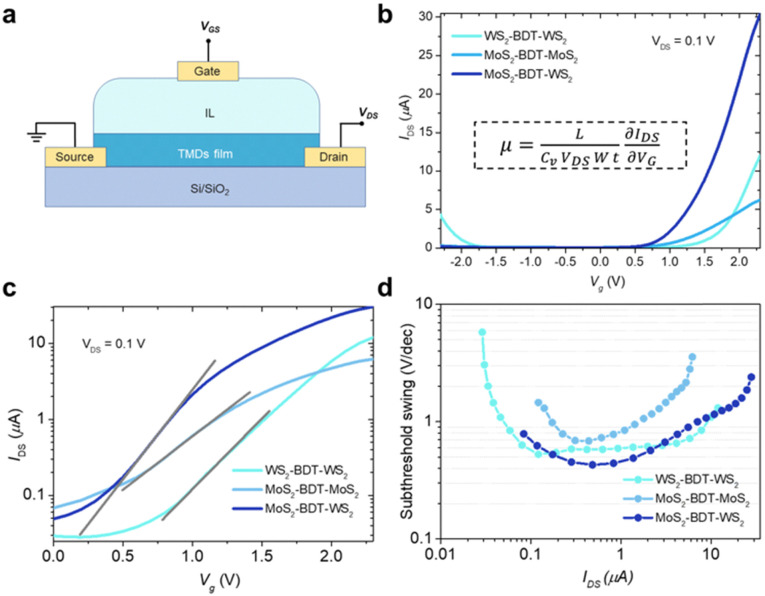
Electrical characteristics of BDT-linked TMD films. (a) Schematic illustration of the FETs. 1-Ethyl-3-methylimidazolium bis(trifluoromethanesulfonyl)imide was readily cast on top of the thin film as ionic liquid (IL). (b) Transfer curves for in-plane BDT-linked TMD films with *V*_g_ sweeping from −2.3 to +2.3 V at *V*_ds_ = 0.1 V. The inset shows the equation for calculating the field-effect mobility, *μ*. *L*, channel length; *C*_v_, volumetric capacitance; *W*, channel width; *t*, film thickness (c) subthreshold slopes obtained from logarithmic-scale current characteristics of BDT-linked TMD films. (d) Subthreshold swing as a function of *I*_DS_ in films based on lateral BDT-linked TMD architectures.

To unveil the electrical characteristics, we have fabricated proof-of-concept bottom-contact, top-gated FETs based on BDT-connected MoS_2_–WS_2_ lateral heterostructures (Fig. 5a and Fig. S24, ESI†). In this device configuration, electrolyte gating induces large carrier densities and enables strong drain-current modulation at low voltage operation.^37,38^ It is worth noting that the choice of an aromatic dithiolated linker over an aliphatic one to yield the covalent network of flakes is critical to grant enhanced inter-flake electronic connectivity and formation of additional percolation pathways, which go beyond the electrical features of films based on defective pristine solution-processed 2D materials.^18,22^ Direct comparison of the electrical performances with films exclusively made of LPE TMDs with similar thickness is not possible as their fabrication in absence of molecular linkers leads to disconnected and rough coatings, inhibiting charge transport across the device (Fig. S15, ESI†), thus exhibiting negligible transistor response. Fig. 5b displays the transfer characteristics of FET devices based on BDT-connected MoS_2_–WS_2_ lateral heterostructure as well as thin films comprising a single 2D material being either MoS_2_ or WS_2_. MoS_2_ and WS_2_ networks, also assembled *via* microfluidic approach, exhibit characteristic n-type and ambipolar behavior, respectively.^12,38,39^ As expected, the lateral heterostructure, composed of a network of alternated MoS_2_ and WS_2_ flakes connected in series by BDT, generates n-type dominated conduction, because only when both MoS_2_ and WS_2_ components are biased to be at ON state, the overall channel is turned ON. Further, the linear and symmetrical output characteristics (Fig. S25, ESI†) suggest that ohmic contact is achieved. Interestingly, the device including the hybrid MoS_2_–WS_2_ lateral heterostructure, exhibiting lower threshold voltage, shows a higher ON current, which gives rise to a higher ON/OFF current ratio. As the formation of MoS_2_–WS_2_ heterojunctions facilitates the electron conduction at the ON state, the fabricated heterostructure yields enhanced field-effect mobility (10^−1^ cm^2^ V^−1^ s^−1^), which outperform of one order of magnitude the mobility values reached by the MoS_2_ and WS_2_ networks (Table S3, ESI†). Importantly, field-effect mobilities obtained in approximately single-flake-thick films by our methodology match state-of-the-art micrometer-thick coatings based on liquid-phase exfoliated TMDs (Table S4, ESI†).^16,18,22^ Therefore, in-plane conductivity in the film is maximized at reduced thickness by molecularly bridging flakes through edge defects, which leads to a continuous thin network without compromising the overall thickness.

To estimate the degree of trap density into semiconducting films, the slope of transfer curves was analyzed (Fig. 5c). The steeper slope of the *I*–*V* characteristics generated by the BDT-linked MoS_2_–WS_2_ heterostructure indicates a lower amount of trap states, which we ascribe to the formation of an intimate network of heterojunctions. To quantitatively assess the trap density (*D*_it_) of the devices, the parameter is extracted from the subthreshold swing (SS, Fig. 5d) by using the following formula:^40,41^1
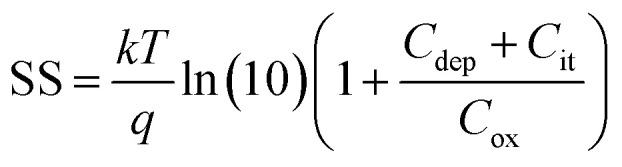
2
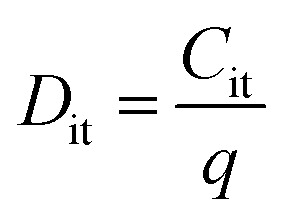
where *k* is the Boltzmann's constant, *T* is the temperature of the measurement, *q* is the electron charge, *C*_ox_ is the oxide capacitance (corresponding to the specific capacitance in IL-gated systems), *C*_dep_ and *C*_it_ are the depletion capacitance and the interface trap capacitance in the weak inversion region, respectively. As the effective substrate bias approaches infinity, *C*_dep_ approaches zero. Thus, the SS can be estimated with the following approximate equation:3
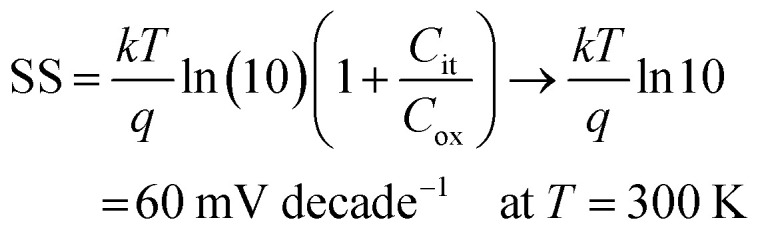
The trap density *D*_it_ in the film based on the lateral heterostructure was calculated to be 3.4 × 10^14^ V^−1^ cm^−2^, being lower than the mono-component BDT-networks with MoS_2_ and WS_2_ which amount to 8.0 × 10^14^ V^−1^ cm^−2^ and 4.4 × 10^14^ V^−1^ cm^−2^, respectively. These results are consistent with the decrease of inter-flake trap density induced by the heterojunction formation in the synthesized MoS_2_–WS_2_ lateral heterostructure.

## Conclusions

In summary, we have demonstrated the planar growth of solution-processed hetero-networks based on 2D TMDs by exploiting intrinsic defects *via* a simple sequential microfluidic approach. Sulfur vacancies in MoS_2_ and WS_2_, mainly located at the edges, have been healed by rigid, bidentate π-conjugated molecules to synergistically anchor adjacent nanosheets and promote the preferential in-plane heterostructure formation, providing percolation across the system at flake density far below state-of-the-art solution-processed films. The fabricated BDT-linked MoS_2_–WS_2_ heterostructures exhibited photophysical features that diverge from conventional blends in the absence of linkers, promoting faster charge separation. Moreover, they are endowed with superior field-effect mobility than mono-component networks due to reduced trap density resulting from the formation of heterojunctions. Our strategy to assemble heterostructures from solution-processed nanosheets can be easily extended to other classes of low-dimensional materials by making use of suitably functionalized bidentate linkers, opening up new avenues in the exploration of unprecedented optoelectronic features that go beyond the current 0D, 1D and 2D materials portfolio. Moreover, the selection of anchoring groups which selectively bind a given nanostructure and cores that can impart novel functionality to the hybrid heterostructure, thereby offering endless combinations for further integrating unprecedented solution-processed low-dimensional assemblies in miniaturized next-generation digital (opto)electronic devices.

## Author contributions

A. G. R. and P. S. conceived the experiments and designed the study. A. G. K. and J. N. C. produced the raw materials. A. G. R. designed and performed the multiscale characterizations on the final functionalized materials. A. G. R. and A. Z. carried out Raman spectroscopy, XPS and impedance analysis. A. G. R. and C. M. performed electrical measurements and interpreted the data. C.E. P. and F. L. designed and performed transient absorption spectroscopy measurements and studies. All authors discussed the results and contributed to the interpretation of data. A. G. R. and P. S. co-wrote the paper with input from all co-authors. All authors have given approval to the final version of the manuscript.

## Data availability

The authors confirm that the data supporting the findings of this study are available within the article and its ESI.†

## Conflicts of interest

There are no conflicts to declare.

## Supplementary Material

MH-011-D4MH00882K-s001
