# Supplementary material for: Defect-engineering of liquid-phase exfoliated 2D semiconductors: stepwise covalent growth of electronic lateral hetero-networks
Source: Mater Horiz. 2024 Sep 6;11(22):5614–21. doi: 10.1039/d4mh00882k (PMC11404671; doi:10.1039/d4mh00882k)
Supplement: MH-011-D4MH00882K-s001 [file MH-011-D4MH00882K-s001.pdf]

Supplementary Information (SI) for Materials Horizons.
This journal is © The Royal Society of Chemistry 2024

Electronic Supplementary Information

Defect-Engineering of Liquid-Phase Exfoliated 2D Semiconductors: Stepwise Covalent Growth of Electronic Lateral Hetero-Networks

Antonio Gaetano Ricciardulli,[a] Christopher E. Petoukhoff, [b] Anna Zhuravlova,[a] Adam G. Kelly,[c] Chun Ma,[a] Frédéric Laquai,[b] Jonathan N. Coleman,[c] and Paolo Samorì[a]*

[a] Université de Strasbourg, CNRS, ISIS UMR 7006, 8 allée Gaspard Monge, 67000 Strasbourg, France

[b] King Abdullah University of Science and Technology (KAUST), Physical Sciences and Engineering Division (PSE), KAUST Solar Center (KSC), Thuwal, 23955-6900, Kingdom of Saudi Arabia

[c] School of Physics, Centre for Research on Adaptive Nanostructures and Nanodevices (CRANN) and Advanced Materials and Bioengineering Research (AMBER), Trinity College Dublin, Dublin 2, Ireland

*Corresponding author: samori@unistra.fr

Table of Contents

		page
1.	Experimental Methods	S3
2.	Controlled experiments on conventional deposition techniques	S7
3.	Optical and morphological investigation	S8
4.	X-ray photoelectron spectroscopy (XPS) analysis	S14
5.	Raman spectroscopy analysis	S17
6.	Electrochemical impedance spectroscopy (EIS)	S21
7.	Transient absorption (TA) spectroscopy analysis	S25
8.	Electrical characteristics in FET based on MoS_2-WS_2 heterostructure	S33
9.	Field-effect mobility comparison	S35
References		S37

1. Experimental Methods

Production of liquid-phase exfoliated MoS_2 and WS_2 inks. MoS_2 and WS_2 inks are obtained by sonicating pristine powders (Sigma Aldrich, purity > 99%) in *N*-Methyl-2-pyrrolidone (NMP) according to a previously reported procedure.[1] The dispersions with initial concentration of 20 mg ml^{-1} in NMP were sonicated for 1 hour using a horn tip sonicator (Sonics Vibra-cell VCX-750 ultrasonic processor) at 60% amplitude and subsequently centrifuged at 3218 g for 1 hour using a Hettich Mickro 220R. The supernatant was discarded to remove potential contaminants from the starting powder. Then, the sediment was redispersed in fresh NMP and sonicated under the same conditions for 5 hours. As a result, polydisperse stock dispersions were yielded, from which flakes can be size selected by stepwise centrifugation. In short, to remove the largest aggregates, the polydisperse stocks are first centrifuged at 106.4 g for 90 min, and the sediment retained for future exfoliation. The supernatant is further centrifuged at 425 g for 90 min to separate the smaller flakes. This sediment is finally redispersed in isopropanol (IPA).

Film formation via microfluidic approach. Si/SiO_2 substrates (15 × 15 mm^2) were rinsed with acetone and 2-propanol to remove any potential contaminant and their surface activated by UV/ozone treatment (NovaScan, Digital UV/Ozone System) for 20 min. Afterwards, to enhance the wettability, the substrates were immersed in 1% 3-(aminopropyl)triethoxysilane (APTES, Alfa Aesar, 98%) solution in water for 30 mins. Then, 0.5 mg mL^{-1} dispersions of MoS_2 and WS_2 in IPA were prepared separately and sonicated for 30 mins in an ice bath. To fabricate the films, a peristaltic pump (Heidolph Hei-Flow Precision 01) was used and IPA dispersions of MoS_2 and WS_2 were alternated. Between each deposition step, a 50 mM solution of 1,4-benzenedithiol (BDT, Sigma Aldrich, ≥ 99%) was circulated. Further details on the film formation are provided in the following SI section, in the text related to Figure S1.

Electrodes fabrication for spectroscopical study. The films were grown on prepatterned substrates to carry out impedance spectroscopy analysis. The electrodes were patterned on a *n*-doped standard Si/SiO_2 (oxide thickness: 90 nm thickness, size: 15 × 15 mm) by maskless photolithography (Microtech LW405B laser writer) using AZ1505 photoresist and MIF726 developer. The channel

length and width were both 10 μm. Then, 3 nm chromium and 40 nm gold were thermally evaporated in turn with high vacuum Plassys MEB 300 followed by sonication-assisted lift-off in acetone. An Olympus BX51 optical microscope was employed to monitor the quality of the lift-off.

Atomic force microscopy (AFM). AFM investigation was performed using a Bruker Dimension Icon operating in air by tapping mode (TESPA-V2 tip, k = 42 N m^{-1}).

Scanning electron microscopy (SEM). SEM images were captured using a FEI Quanta 250 FEG scanning electron microscope operated in high vacuum mode (10^{-4} Pa) and with accelerating voltages of 30 kV for the incident beam.

X-ray photoelectron spectroscopy (XPS). XPS analysis was carried out with a Thermo Scientific K-Alpha X-ray photoelectron spectrometer, equipped with a chamber pressure of ~10^{-9} mbar and an Al anode X-ray source (1486 eV radiation). Spot sizes of 400 μm and pass energies of 200.00 eV for wide energy scans and 20.00 eV for high-resolution scans were used on fabricated films based both on pristine materials and lateral heterostructures, respectively. The peak fitting of high-resolution XPS spectra S2p, Mo3d and W4f regions was performed using characteristic doublets with the following fitting constraints: i) S2p, L/G Mix = 30%, spin-orbit splitting = 1.20 ± 0.1 eV and intensity ratio = 0.511 for S2p3/2 and S2p 1/2 components; ii) Mo3d, L/G Mix = 30%, spin-orbit splitting = 3.15 (+0.2-0.1) eV and intensity ratio = 0.690 for Mo3d5/2 and Mo3d3/2 components; iii) W4f, L/G Mix = 30%, spin-orbit splitting = 2.15 ± (+0.2-0.1) eV and intensity ratio = 0.788 for W4f7/2 and W4f5/2 components. For quantification, corresponding atomic scattering factors were extracted from quantification library of the Avantage software package and automatically corrected for the instrument transmission function.

Raman spectroscopy. Raman spectroscopy measurements were performed with a Renishaw inVia spectrometer at 532 nm, which is equipped with a 2D-CCD camera. To avoid sample damages, the films were irradiated with laser power ≤ 1% and exposure times ≤ 10 s.

Electrochemical impedance spectroscopy (EIS). EIS measurements in NaCl aqueous solution, used as electrolyte, were carried out operating a Metrohm Autolab PGSTAT204 potentiostat coupled with a mini probe station (Everbeing Int'l Corp.). EIS tests were performed from high to low frequencies, using a frequency range of 0.1 - 10^5 Hz and a sine-wave voltage signal amplitude of 50 mV (root-mean-square, RMS). Data was analyzed with the software NOVA, Metrohm Autolab.

Electrical characterizations. The field-effect transistors (FETs) performance characteristics were measured by a software-controlled source meter (Keithley 2636A) at room temperature and under nitrogen atmosphere into a glovebox. A platinum wire and a droplet of 1-ethyl-3-methylimidazolium bis(trifluoromethylsulfonyl)imide (EMI-TFSI), purchased from TCI Chemicals, were used as the gate electrode and the ionic liquid gate dielectric, respectively. Thin films for electrical characterizations were built bottom-contact *n*-doped Si/SiO_2 substrates (15 × 15 mm; Fraunhofer IPMS), consisting of thermally grown SiO_2 (230 nm thick) with interdigitated gold electrodes spaced at 2.5 μm and channel length of 10 mm.

Transient absorption (TA) spectroscopy. TA spectroscopy was carried out using a home-built pump-probe setup. The output of a Ti:sapphire amplifier (Coherent LEGEND DUO, 800 nm, 4.5 mJ, 3 kHz, 100 fs) was split into four beams (2 mJ, 1 mJ, 1 mJ, and 0.5 mJ). Three of them were used to separately pump three optical parametric amplifiers (OPA; Light Conversion TOPAS Prime). One of the 1 mJ TOPAS was used to generate wavelength-tunable pump pulses (240-2600 nm, using Light Conversion NIRUVIS extension), which was fixed at 500 nm with a fluence of 9.5 $\mu J/cm^2$. The second 1 mJ TOPAS was used to generate signal and idler only (1160-2600 nm), which was fixed to 1300 nm and was used as the seed for white light generation by focusing a portion of it through a continuously moving CaF_2 crystal, thereby generating a white light

supercontinuum from 350 nm to 1100 nm. The pump-probe delay time was achieved by varying the probe path length using a broadband retroreflector mounted on a 600 mm automated mechanical delay stage (Thorlabs optical delay line ODL600/M), generating delays from -400 ps to 7 ns.

Pump and probe beams were focused on the sample to spot sizes of 0.704 mm and 0.049 mm average diameter (from a Gaussian fit at 86.5% intensity), as measured using a beam profiler (Coherent LaserCam-HR II). The samples were kept under a dynamic vacuum of $<10^{-5}$ mbar, and pump and probe beams were incident on the film-side of the sample. The transmitted fraction of the white light was guided to a custom-made prism spectrograph (Entwicklungsbüro Stresing) where it was dispersed by a prism onto a 512-pixel NMOS linear image sensor (Hamamatsu S8381-512Q). The probe pulse repetition rate was 3 kHz, while the excitation pulses were mechanically chopped to 1.5 kHz, and the detector array was read out at 3 kHz. Adjacent diode readings corresponding to the transmission of the sample after excitation and in the absence of an excitation pulse were used to calculate ΔT/T. Measurements were averaged over several thousand shots to obtain a good signal-to-noise ratio. The chirp induced by the transmissive optics was corrected with a home-built Matlab code.

To account for the spectral shifts of the excited state signals, TA spectra were fit with a sum of either 1, 2, or 3 Gaussians at every pump-probe delay time to determine the peak shifts (**Figure S22**). Kinetic traces of each exciton feature were obtained by taking the maximum ΔT/T value at the wavelength determined from the Gaussian fit for each species to account for the spectral shifts. (**Figure S23**). The kinetics were fitted with a biexponential decay equation: $\Delta T/T = A_0 + A_1 e^{\left(\left(-t/\tau_1\right)\right)} + A_2 e^{\left(\left(-t/\tau_2\right)\right)}$, where t is the pump-probe delay time, A_i are the amplitudes of the decay components, and t_i are the lifetimes of the decay components, and the average lifetimes (t_{ave}) were calculated by taking the amplitude-average of the fast and slow components.

2. Controlled experiments on conventional deposition techniques

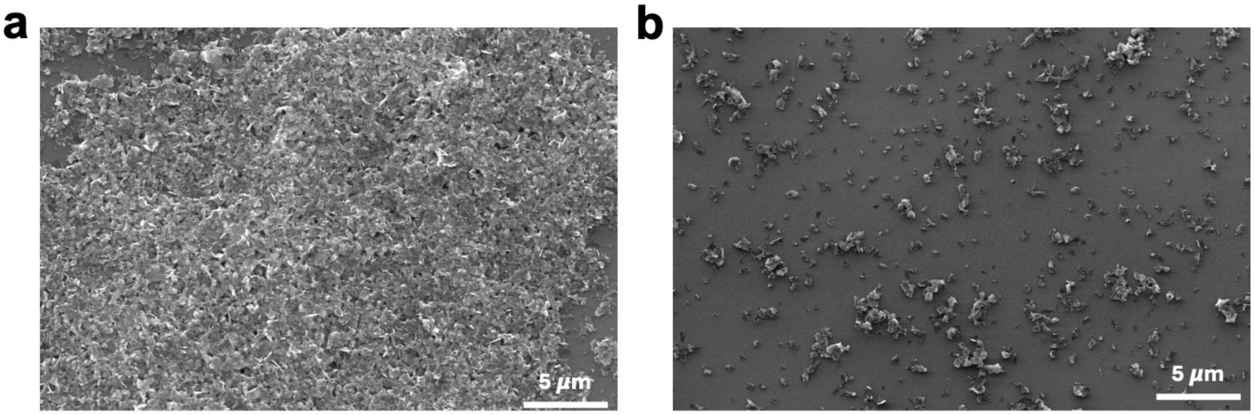

Figure S1. SEM images on deposition of LPE TMDs flakes through (a) drop-casting and (b) spin-coating. As a result of the lack of assembly control, the deposited films display aggregated flakes and uneven coverage, hindering the use of alternating flakes to build lateral hetero-networks.

3. Optical, morphological and topographical investigation

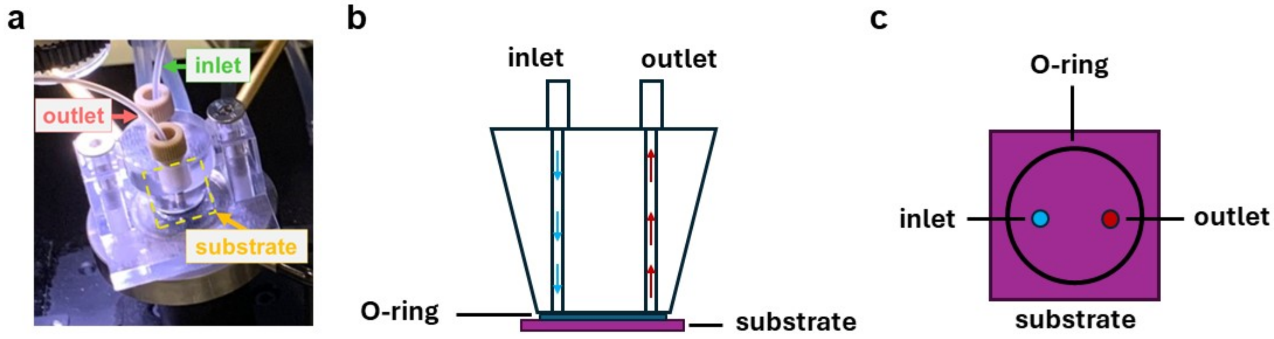

Figure S2. (a) Optical image of the microfluidic chamber; schematic illustration of the microfluidic setup: (b) side- and (c) top-view.

The films were growth stepwise using a microfluidic setup including a microfluidic chamber (Figure S1) with an internal volume of ~12 μL and a peristaltic pump. The optimal flow rate of 0.6 mL min^{-1} was kept constant throughout the deposition. Moreover, a tube diameter of 0.8 mm was employed to avoid aggregation of flakes during the fabrication process, which can lead to clogging, and thus to uneven coatings. First, the substrate was placed in the chamber and coated with MoS_2 by flowing the dispersion of MoS_2 in IPA for 3 minutes. This step yielded randomly distributed MoS_2 flakes on the surface (Figure S3). In contrast, exposure times lower or higher than 3 minutes led to scarce or dense MoS_2 distribution, respectively. Then, a 50 mM solution of BDT in IPA was circulated into the system for 2 minutes to trigger the functionalization at the MoS_2 defect sites. Afterwards, a dispersion of WS_2 in IPA was used for 10 minutes to enable the linking with free thiols terminals, granting linking through defects and formation of a first MoS_2-BDT-WS_2 in-plane network. Subsequently, BDT was used as described before and the previous steps repeated to yield a lateral heterostructure. It worth noting that between each step, the whole system was flushed with IPA to clean the tubes and chamber by a possible excess of material and remove any TMD flake or BDT molecules that did not attach onto the surface of the substrate or where covalently tethered to the forming network. An overview of further processing parameters that have been controlled, such as flow rate, tube diameter, dispersions concentration, is provided in Figure S4.

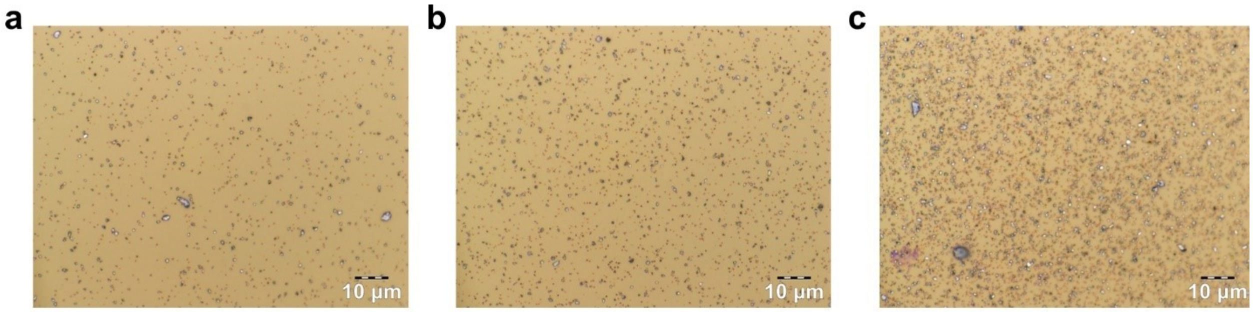

Figure S3. Optical images of Si/SiO_2 substrate coated with MoS_2 after (a) 2, (b) 3 and (c) 5 minutes of MoS_2 dispersion flow.

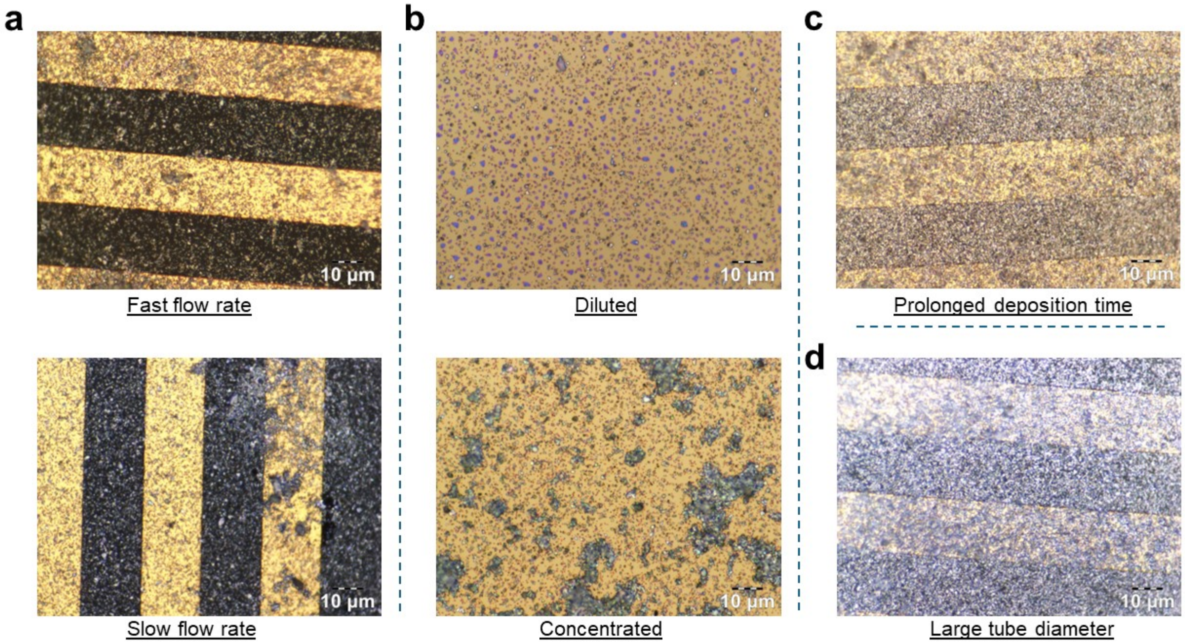

Figure S4. Processing parameters control. (a) Flow rates diverging from the optimal value of 0.6 mL min^{-1} led to uneven coatings, as observed by using rates of 1.0 mL min^{-1} (top) and 0.2 mL min^{-1} (bottom), respectively. (b) Concentrations of as-synthesized MoS_2 and WS_2 inks, above 1 mg mL^{-}1 led to the formation of aggregated clusters of TMDs and inhomogeneous coatings (bottom). In contrast, the diluted counterparts (0.1 mg mL^{-1}) yielded scarce coverage (top). The optimal dispersion concentration in the microfluidic approach is 0.5 mg mL^{-1}. The use of longer deposition times (c) or (d) large tube diameter (d), such as 1.2 mm, produced irregular and rough films.

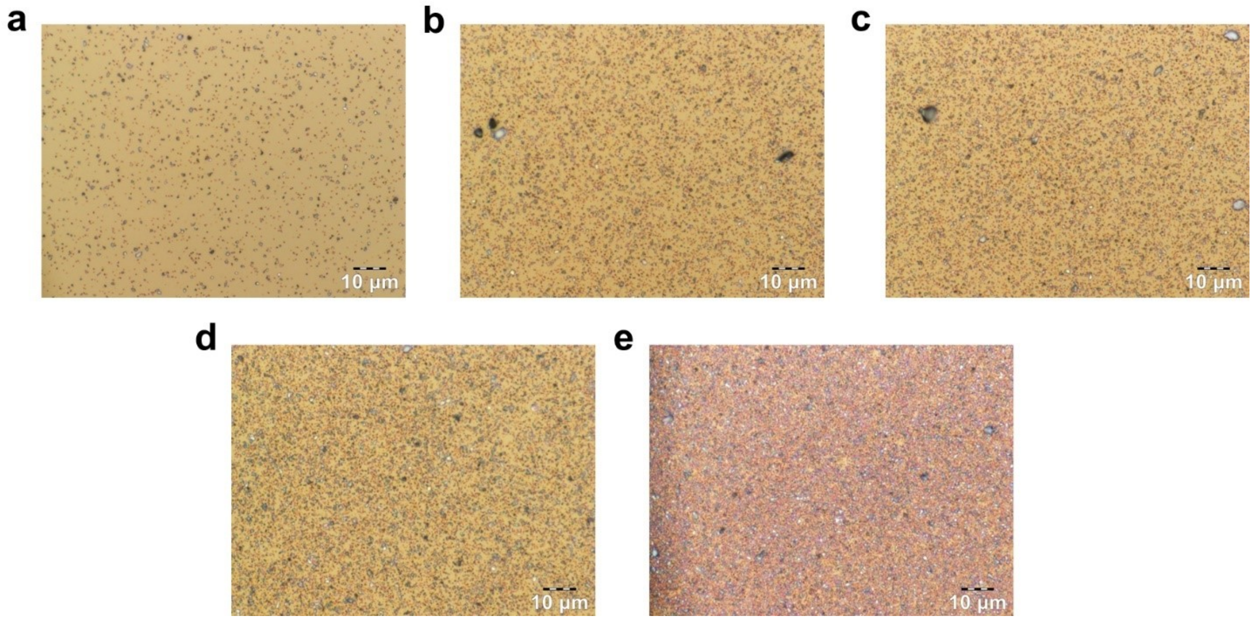

Figure S5. Optical microscopy survey images of the in-plane growth of the MoS_2-WS_2 heterostructure assisted by BDT bridging. Corresponding film after the stepwise deposition of (a) MoS_2, (b) WS_2, (c) MoS_2, (d) WS_2 and (e) MoS_2. BDT solution in IPA was circulating after between steps.

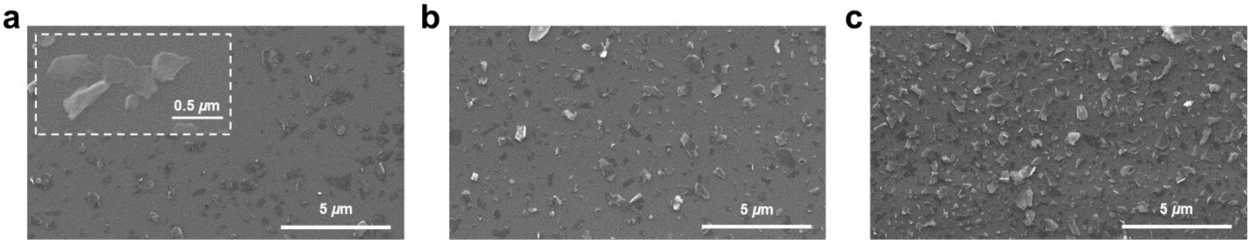

Figure S6. SEM images of the intermediate steps of the lateral heterostructure corresponding to (a) Mo_2-WS_2 (inset: in-plane junction within flakes), (b) MoS_2-WS_2-MoS_2 and (c) MoS_2-WS_2-MoS_2-WS_2.

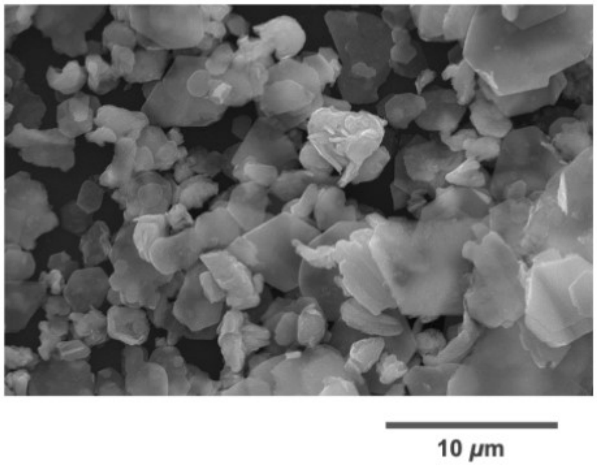

Figure S7. SEM image of a film based on MoS_2-WS_2 hetero-network after further deposition steps exceeding the optimization threshold, which result in undesirable aggregations and vertical stacking of flakes.

4. X-ray photoelectron spectroscopy (XPS) analysis

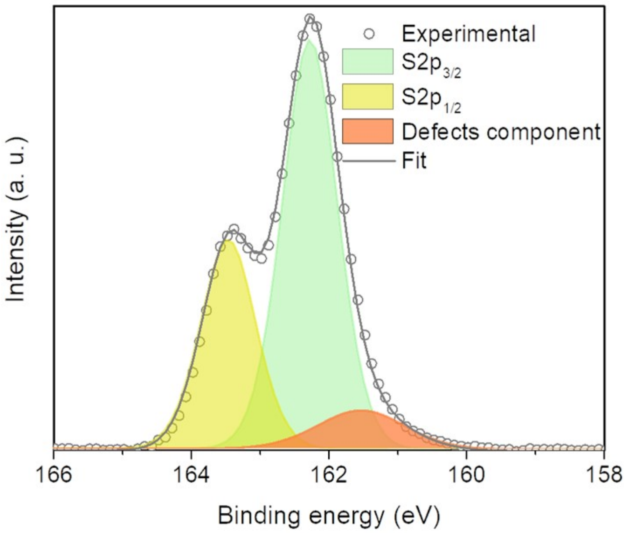

Figure S8. High-resolution (HR) XPS spectrum of the S 2p region in pristine MoS_2-WS_2 blend.

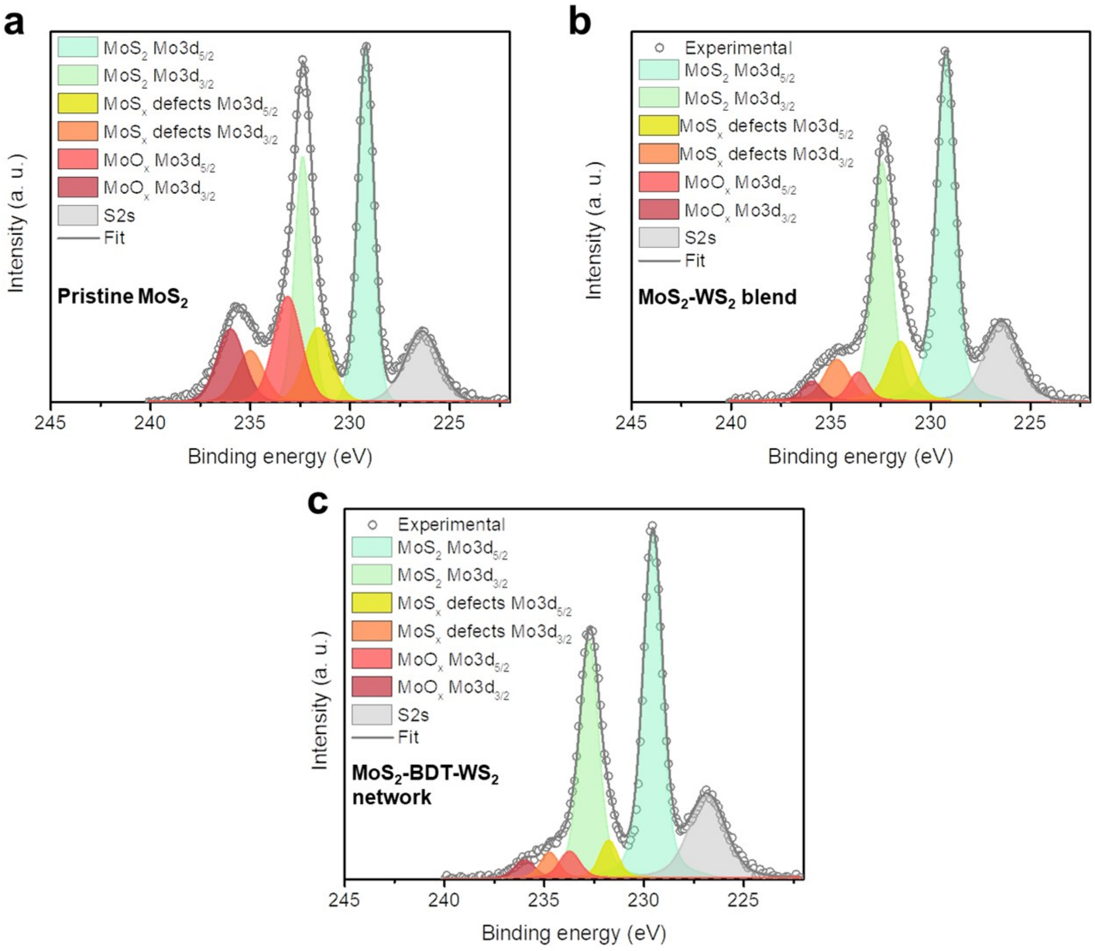

Figure S9. HR-XPS spectra of Mo 3d peak in (a) pristine MoS_2, (b) MoS_2-WS_2 blend and (c) MoS_2-WS_2 BDT-linked lateral heterostructure.

The Mo 3d region of MoS_2, pristine MoS_2-WS_2 blend and MoS_2-BDT-WS_2 lateral heterostructure is characterized by a set of doublets emerging from the Mo $3d_{5/2}$ and Mo $3d_{3/2}$ spin-orbital splitting (Figure S9). The intense characteristic MoS_2 doublet appears at ~229.3 and ~232.5 eV. In contrast, the peaks at ~233.4 and ~236 eV correspond to oxidized Mo species. Moreover, the defect component can be deconvoluted at ~231.5 and ~234.7 eV. The S2s singlet is displayed at ~226.6 eV. The significant decrease of the relative area of the defect component from 16% in pristine MoS_2 and 15% in the pristine MoS_2-WS_2 blend to 8.6% in the MoS_2-BDT-WS_2 network suggests the successful functionalization by BDT through sulfur vacancies healing.[2] These results further support the evidence of the healing process confirmed by the HR-XPS analysis of S 2p region.

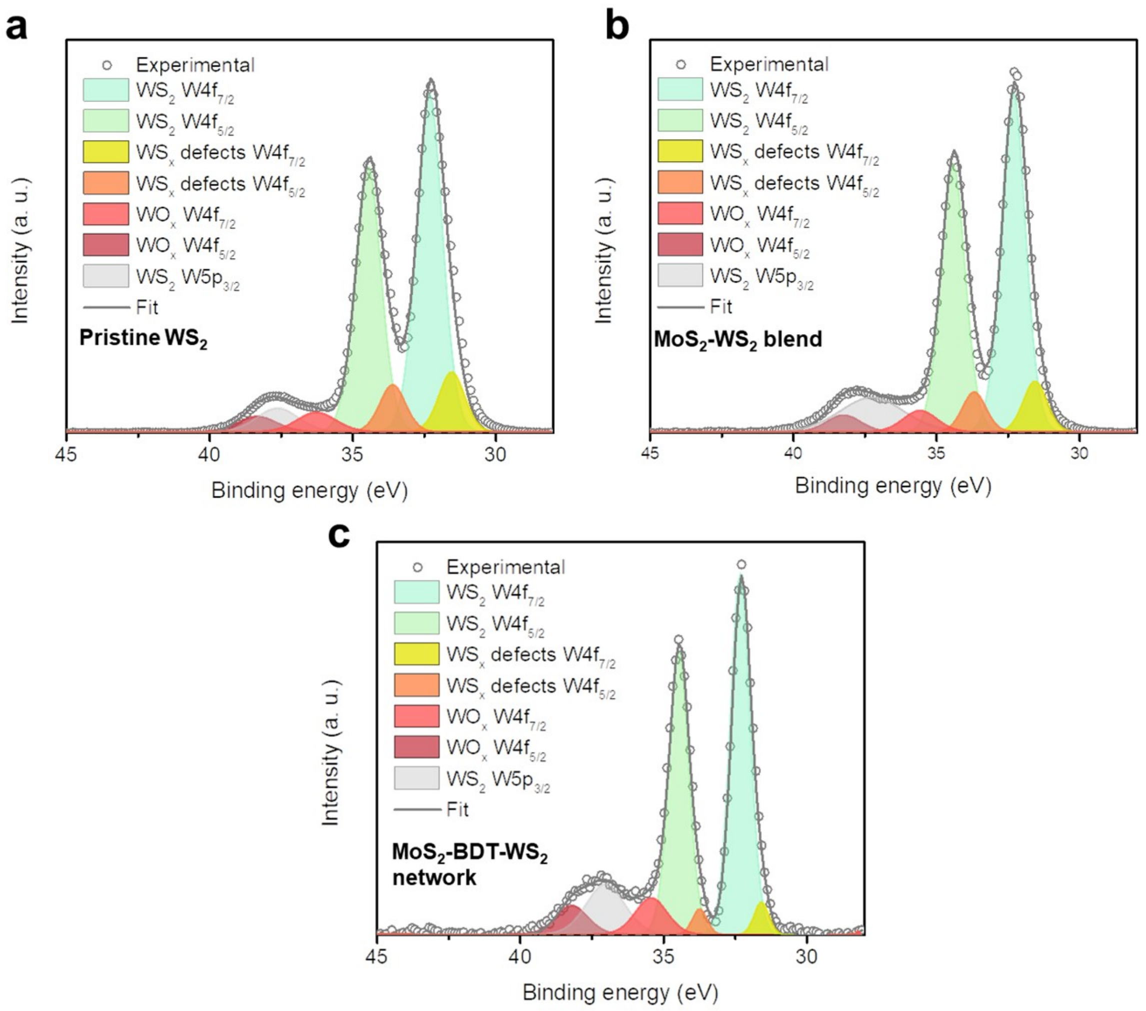

Figure S10. HR-XPS spectra of W 4f peak in (a) pristine WS_2, (b) MoS_2-WS_2 blend and (c) MoS_2-WS_2 BDT-linked lateral heterostructure.

The W 4f region features a set of doublets due to the W $4f_{7/2}$ and W $4f_{5/2}$ spin-orbital splitting (Figure S9). The intense WS_2 peaks lay at ~32.3 and ~34.5 eV. The oxidized tungsten impurity is present at ~36 and ~38.3 eV. The defect component of WS_2 emerges at ~31.5 and ~33.7 eV. In addition, this energy region is characterized by a W $5p_{3/2}$ peak at ~37.2 eV. As for MoS_2 (Figure S10), the relative area of the defect component in WS_2 decreases from 18% for pristine WS_2 and 15% for the MoS_2-WS_2 blend to 7% in the MoS_2-BDT-WS_2 film. These results further prove the successful healing of defects in WS_2 through BDT functionalization,[3] as also supported by the reduction of the defect density displayed in the S 2p region.

5. Raman spectroscopy analysis

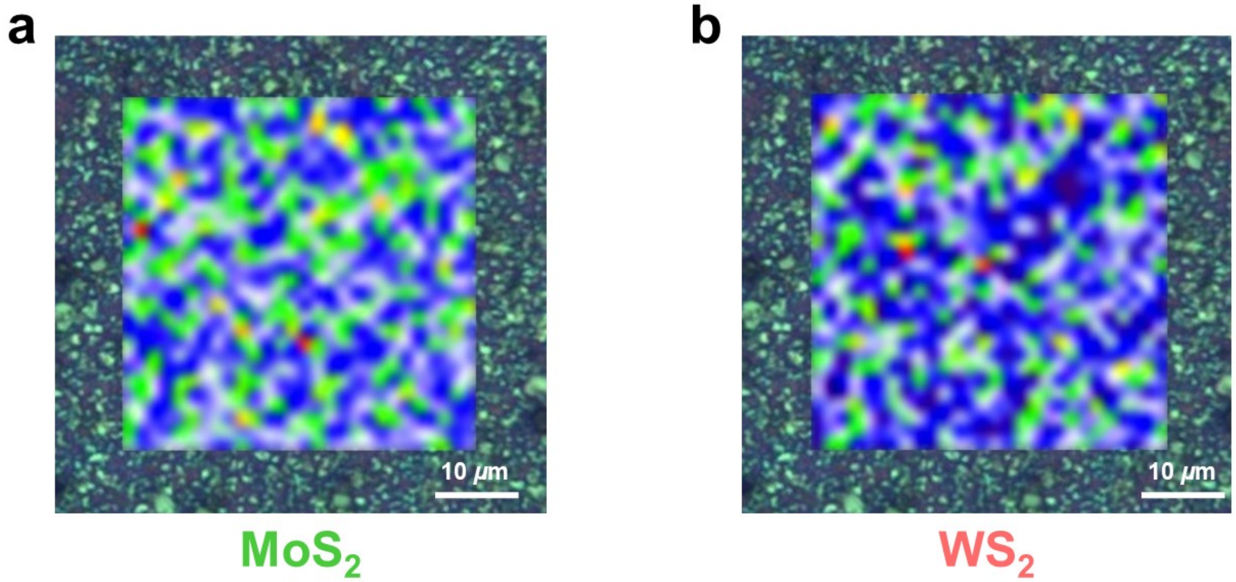

Figure S11. Raman mapping of (a) MoS_2 and (b) WS_2 building units in the BDT-linked heterostructure.

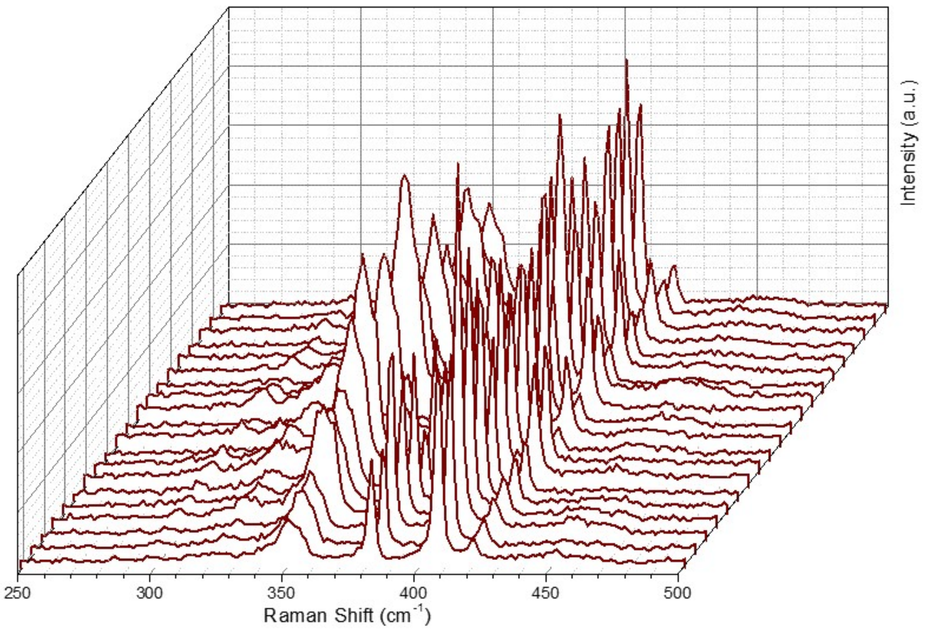

Figure S12. Random selected Raman spectra of the MoS_2-BDT-WS_2 hetero-network showing the characteristic bands of both MoS_2 and WS_2, revealing equal distribution of the 2D building units. Furthermore, the characteristic Raman features of MoS_2 and WS_2 are retained, indicating that the BDT does not induce any apparent change to the intrinsic structural features of the TMDs.

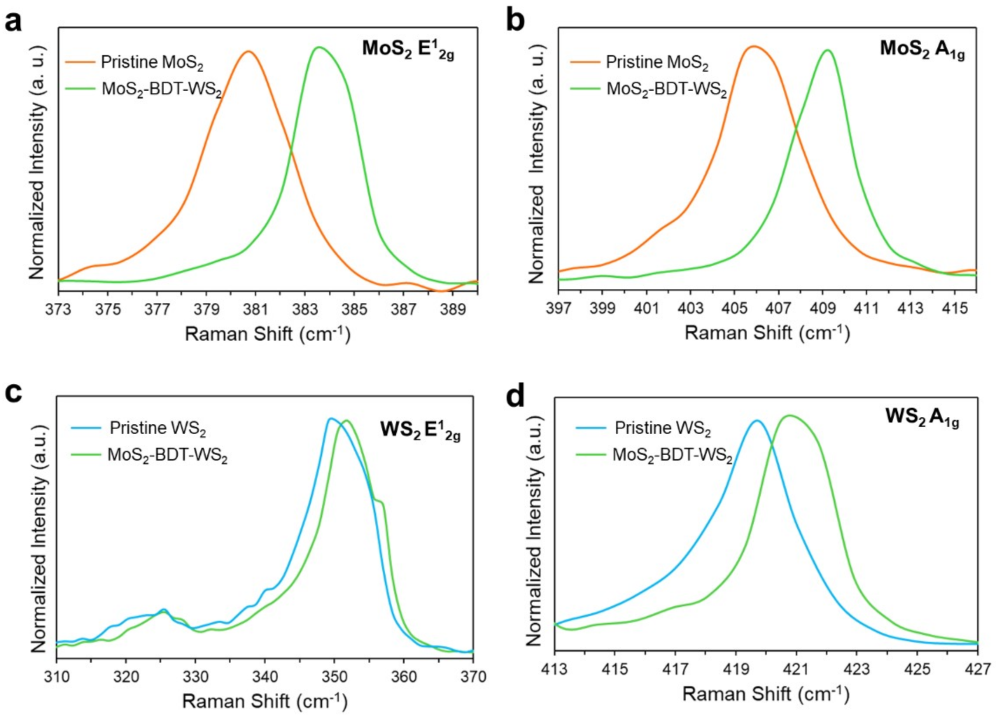

Figure S13. Comparison of (a) E^1_{2g} and (b) A_{1g} Raman peaks of MoS_2 in pristine LPE MoS_2 film and MoS_2-BDT-WS_2 lateral heterostructure. (c) E^1_{2g} and (d) A_{1g} Raman peaks of WS_2 in pristine LPE WS_2 film and MoS_2-BDT-WS_2 heterostructure.

A significant blue shift is observed for E^1_{2g} and A_{1g} peaks, related to in-plane and out-of-plane vibrations, respectively, for both MoS_2 and WS_2 in the MoS_2-BDT-WS_2 film (Figure S13). Such variations are in agreement with the suppression of defects-activated modes upon BDT healing.[4] As the coverage of the MoS_2-BDT-WS_2 heterostructure is larger than the one of both pristine films and simple blend, the flake-substrate interaction at the interface is critical. Thus, the out-of-plane mode tends inevitably to be stabilized as the nanosheets approach the surface of the substrate, leading to a significant blue shift in both the A_{1g} components of MoS_2 and WS_2. Additionally, cross-linking with BDT plays a significant role in the system rigidity, further constraining the out-of-plane mode. Moreover, the frequency of the E^1_{2g} mode is strictly correlated with defect density. Hence, a blue shift of the vibrational frequencies occurs upon decrease of defect density.[4] Further evidence is provided by the FHWM contraction that occurs for the E^1_{2g} and A_{1g} features of both MoS_2 and WS_2 in the heterostructure built by employing BDT as linker (Table S1).

Material	FWHM E^1_{2g} (cm^{-1})	FWHM $A1_g$ (cm^{-1})
MoS_2	4.20 ± 0.25	4.36 ± 0.25
MoS_2 (MoS_2-BDT-WS_2)	2.82 ± 0.48	3.45 ± 0.49
WS_2	11.39 ± 0.37	3.35 ± 0.15
WS_2 (MoS_2-BDT-WS_2)	10.63 ± 0.24	3.01 ± 0.23

Table S1. Statistical analysis over 20 Raman spectra of FWHM of E^1_{2g} and A_{1g} peaks of pristine MoS_2, pristine LPE WS_2 and MoS_2-BDT-WS_2 films.

6. Electrochemical impedance spectroscopy (EIS)

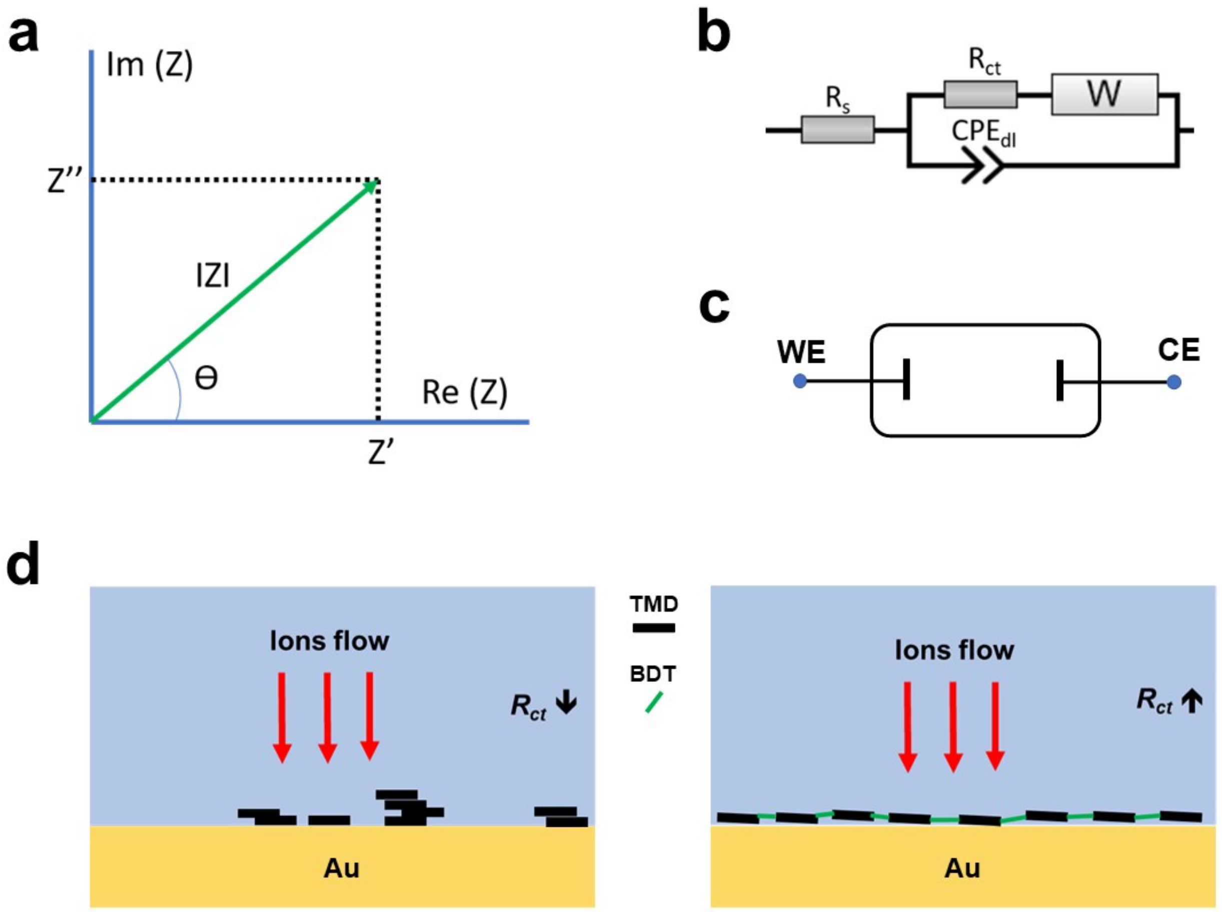

Figure S14. (a) Schematic representation of the impedance Z. Z' and Z'' are real and imaginary components of Z, θ is the phase and $|Z|$ is the modulus. (b) Schematic illustration of the Randle's circuit that is used in this study. (c) Scheme of the symmetrical two-electrode setup. (d) Schematic illustration of ionic charge transfer process for pristine LPE TMD-based film (left) and TMD lateral heterostructure.

EIS is a powerful tool for the study of thin films and interfaces. EIS relies on the small amplitude sinusoidal potential or current perturbations at different frequencies.[5,6] Accordingly, the sinusoidal response of current or potential is collected and translated to the electrochemical impedance of the cell at a given frequency:

$$Z(\omega) = \frac{\tilde{V}(\omega)}{\tilde{I}(\omega)} \tag{1}$$

$$Z(\omega) = \left|\frac{\tilde{V}(\omega)}{\tilde{I}(\omega)}\right|(cos\varphi(\omega) + isin\varphi(\omega)) = Z^{'} + iZ^{''} \tag{2}$$

$$|Z| = \sqrt{(Z^{'})^2 + (iZ^{''})^2} \tag{3}$$

where ω is the angular frequency and can be expressed through the frequency f as $\omega = 2\pi f$, φ is the phase angle between the input and the response signals, $\tilde{V}$ and $\tilde{I}$ are phasors of the corresponding sinusoidal functions. The overall impedance Z is frequency-dependent and can be expressed as a sum of its real ($Z^{'}$) and imaginary ($Z^{''}$) parts (1,2),[6] while the modulus of the impedance is calculated as the square root of the squared $Z^{'}$ and $Z^{''}$ (3).[7] The graphical representation of the impedance and its components is given in Figure S11a. Typically, EIS results are represented in the form of Nyquist plots where the *x* and *y* axes are $Z^{'}$ and $Z^{''}$, respectively. These diagrams allow the graphical separation of the processes that occur in the electrochemical cell. Ultimately, every system can be modelled with an equivalent circuit composed of primitive circuit elements. Each element is associated with a real process in the cell. The Randle's equivalent circuit, which comprises solution resistance R_s, charge transfer resistance R_{ct}, double layer capacitance CPE_{dl} and diffusion-related Warburg impedance *W*, was employed in this study (Figure S11b). Such model provides direct comparison among all the dataset with minimized error.

The experimental setup (Figure S12c) consists of a symmetrical two-electrode system that is composed of the identical interdigitated working electrode (WE) and counter electrode (CE), a TMD film that grown stepwise on top of WE and CE, and aqueous electrolyte. Thus, the measured impedance includes all the contributions of the processes between WE and CE.[6] In such system, the ions migrate from the electrolyte towards the electrodes upon the perturbation of the potential, generating ion conductance. The TMD film, formed between the electrodes and the electrolyte, acts as a barrier to the ion flow, hindering the charge transfer at the interface. Therefore, the lateral and uniform growth of the film leads to a considerable R_{ct} increase (Figure S11d).

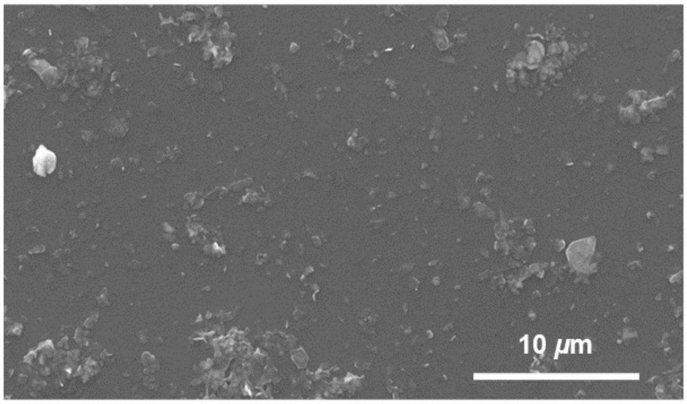

Figure S15. SEM image of a film fabricated by alternating MoS_2 and WS_2 multiple times without using BDT as intermediate step.

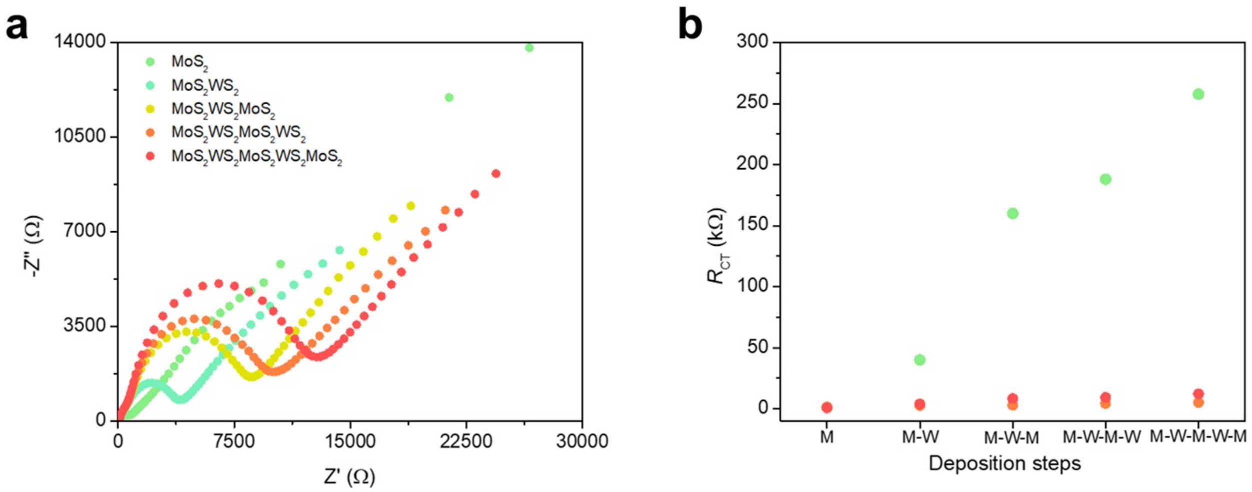

Figure S16. (a) EIS signal evolution upon increase of MoS_2 and WS_2 deposition. Between each step, an IPA solution of thiophenol is circulated. (b) R_{ct} increase after each MoS_2 and WS_2 deposition step in films with BDT (green), thiophenol (red) and without any molecular linker (orange).

7. Transient spectroscopy (TA) analysis

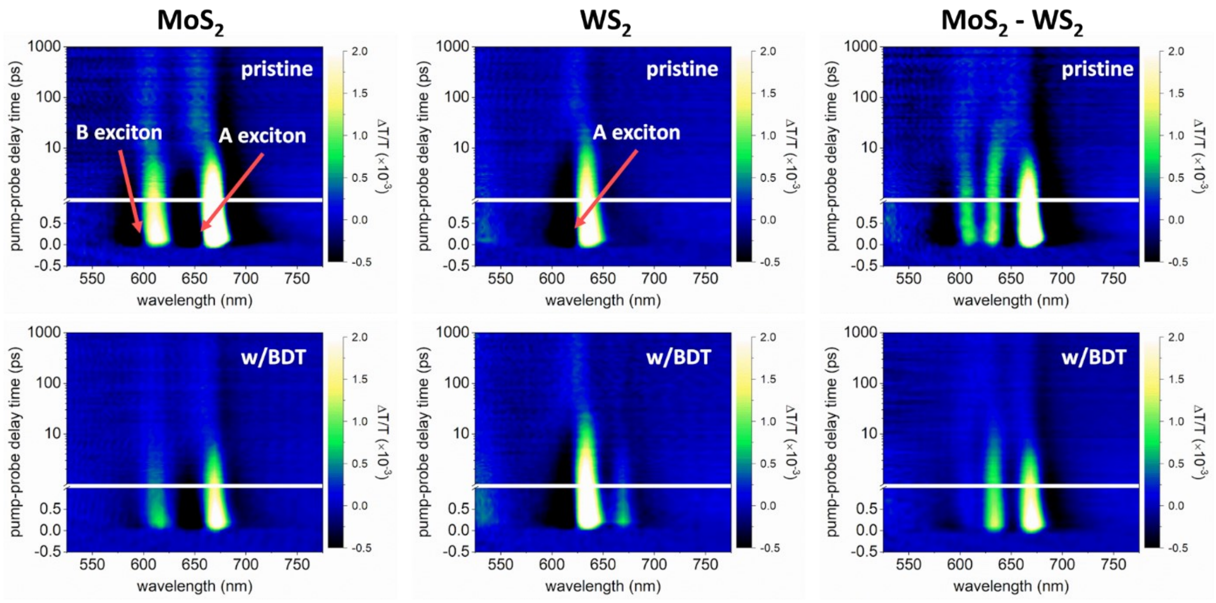

Figure S17. Contour plots of transient absorption (TA) spectra of the pristine MoS_2, WS_2, and MoS_2-WS_2 heterostructures (top row), and the samples treated with BDT (bottom row).

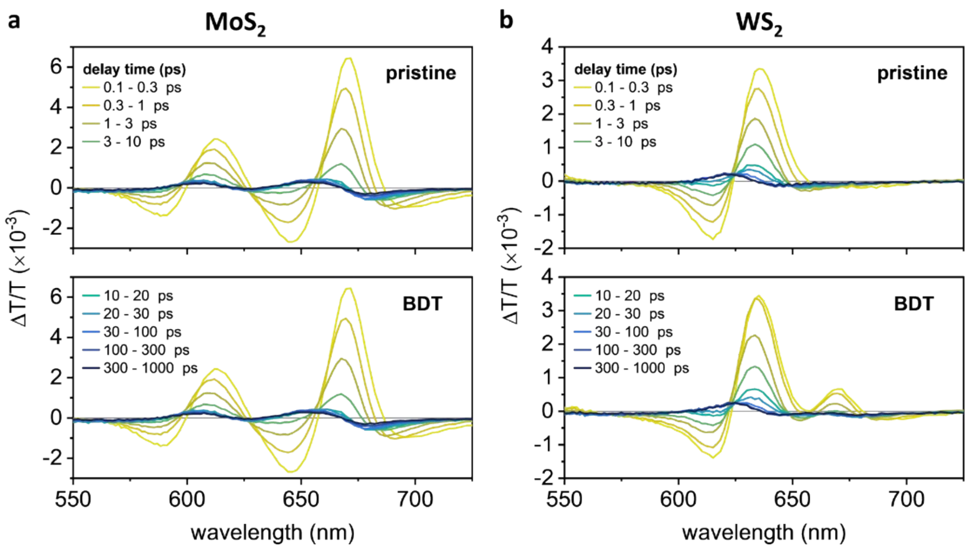

Figure S18. Transient absorption (TA) spectra of the isolated TMDs with and without BDT: (a) MoS_2 and (b) WS_2. The spectra are averaged over the pump-probe delay times listed in the legend.

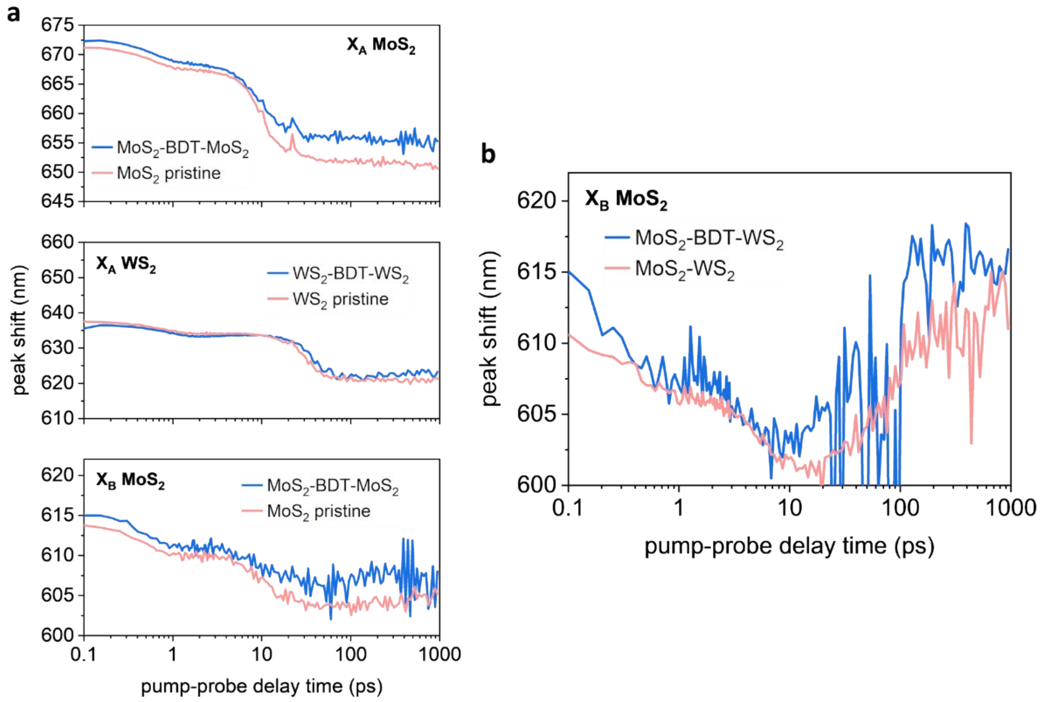

Figure S19. Peak shifts for the reported species, with or without BDT, as determined from Gaussian fits to the individual peaks, for: (a) isolated TMDs: X_A of MoS_2 (top); X_A of WS_2 (middle); X_B of MoS_2 (bottom); or (b) X_B of MoS_2 in the MoS_2-WS_2 heterostructures.

All the photoexcited species within the neat TMD materials, with or without BDT, blue-shifted with increasing pump-probe delay time, due to their bandgaps returning to their ground-state values (**Figure S19a**). X_B of MoS_2 in the heterostructures, however, exhibited different behavior from the pristine materials (**Figure S19b**). The peaks blue-shifted up to ~10-20 ps, at which point they again red-shifted. We attribute this to competition between relaxation of the bandgap renormalization at faster timescales with the slow transfer of trapped carriers at longer timescales.[8]

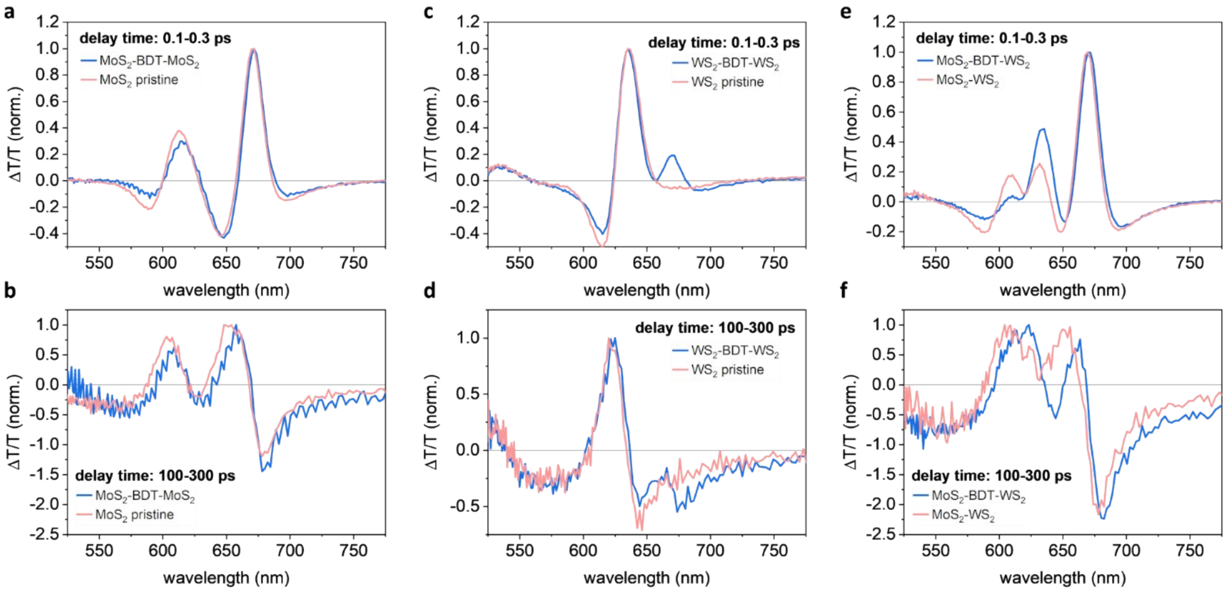

Figure S20. Transient absorption (TA) spectra of MoS_2 (a,b), WS_2 (c,d), and MoS_2-WS_2 heterostructures (e,f) with and without BDT. The spectra are averaged over early delay times (a,c,e: 0.1-0.3 ps) or late delay times (b,d,f: 100-300 ps).

We note that X_A and X_B of MoS_2 linked with BDT were consistently red-shifted both for the pristine films (MoS_2-BDT-MoS_2) and the heterostructures (MoS_2-BDS-WS_2) for all delay times (**Figure 4c** and **Figure S19**). To investigate this in more detail, we compared the TA spectra of pristine and BDT-linked TMDs at different pump-probe delay times (**Figure S20**). At late delay times (i.e., 100-300 ps), after the TMD bandgaps returned to their ground state values, the spectral peaks should match the ground state differential transmission spectra. For both neat MoS_2 (**Figure S20b**) and the MoS_2-WS_2 heterostructure (**Figure S20f**), the TA spectra at 100-300 ps were red-shifted in the presence of BDT compared to the pristine TMDs. Red-shifts of the excitonic peaks of MoS_2 have been attributed to reduction in the exciton binding energy [R],[8] which suggests that the BDT molecule enables easier separation of the exciton compared to the unlinked heterostructure. We do not observe such red-shifts in X_A of neat WS_2 in the presence of BDT at almost any delay time (**Figure S20c,d** and **Figure S19a**), suggesting that the BDT does not have as large of an effect on the exciton binding energy of WS_2 alone.

This is in stark contrast to the X_A of WS_2 in the heterostructure, which exhibits completely different spectral shifts depending on either the formation of an interlayer exciton (MoS_2-WS_2) or charge separation (MoS_2-BDT-WS_2), as discussed in the main text (**Figure 4d**).

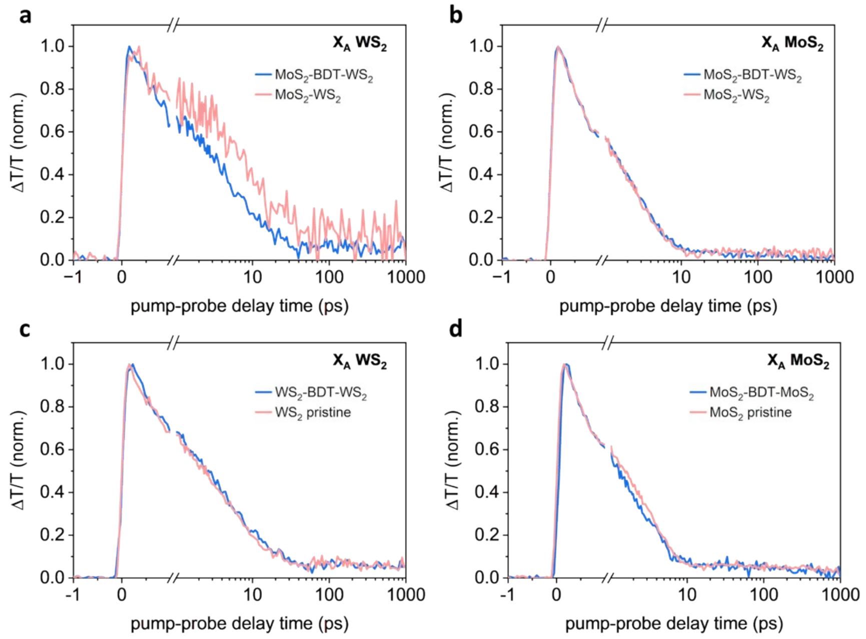

Figure S21. Transient absorption kinetic traces of the X_A of WS_2 (a, c) and the X_A of MoS_2 (b, d) within either the MoS_2-WS_2 heterostructures (a, b) or the isolated monolayers (WS_2 or MoS_2; c, d, respectively) with or without BDT.

The BDT-linked heterostructures exhibited faster relaxation dynamics compared to the unlinked heterostructures (**Figure S21**). X_A of WS_2 in the unlinked MoS_2-WS_2 heterostructure had a much longer lifetime (7.7 ps) compared to the BDT-linked heterostructure (4.5 ps; **Figure S21a** and **Table S2**). This longer lifetime further supports formation of tightly bound interlayer excitons within the unlinked heterostructures, as described in the main text.[9] The interlayer excitons were not likely present for the BDT-linked heterostructures such that charge separation could thus proceed at a much faster rate. We note that this effect was most pronounced for the X_A WS_2 excitons; X_A of MoS_2 had nearly identical lifetimes with or without BDT (~2 ps; **Figure S21b** and **Table S2**). We further note that the lifetime of the X_A WS_2 and X_A MoS_2 excitons were unchanged in the isolated monolayer films with or without BDT (**Figure S21c, d** and **Table S2**). The long-lived species present in all samples at time scales >30 ps comes from trapped excitons,[10] which was significantly higher for the unlinked heterostructure (up to 11.8% for X_A WS_2) compared with the BDT-linked one (up to 5.7% for X_A WS_2) (**Table S2**), further demonstrating the reduction of sulfur vacancies upon BDT functionalization.

**Sample**	**Species**	**A_0 (%)**	**A_1 (%)**	**t_1 (ps)**	**A_2 (%)**	**t_2 (ps)**	**t_{ave} (ps)**
MoS_2	X_A (MoS_2)	4.40	30.22	0.490	65.38	2.99	2.10
MoS_2-BDT-MoS_2	X_A (MoS_2)	4.10	53.79	0.968	42.10	3.67	2.06
MoS_2-WS_2	X_A (MoS_2)	3.06	36.14	0.626	60.79	2.82	1.94
MoS_2-BDT-WS_2	X_A (MoS_2)	2.09	41.64	0.618	56.26	3.30	2.11
WS_2	X_A (WS_2)	5.97	41.00	0.805	53.03	7.08	4.09
WS_2-BDT-WS_2	X_A (WS_2)	5.65	42.25	0.846	52.10	8.00	4.53
MoS_2-WS_2	X_A (WS_2)	11.78	21.57	0.532	66.65	11.35	7.68
MoS_2-BDT-WS_2	X_A (WS_2)	5.74	36.44	0.678	57.81	7.34	4.49

Table S2. Summary of fits of kinetic traces.

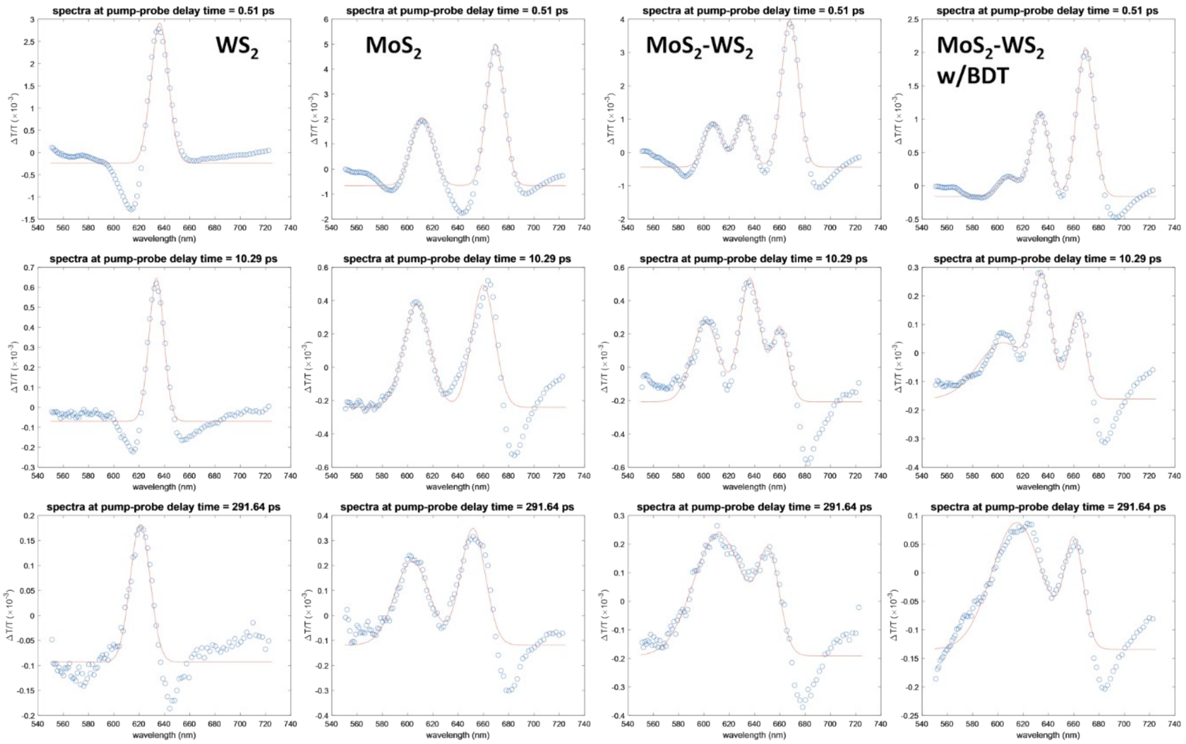

Figure S22. Examples of multi-Gaussian fits for the TA spectra at different pump-probe delay times. 1 Gaussian was used for WS_2; 2 Gaussians for MoS_2; and 3 Gaussians were initially used for the MoS_2-WS_2 heterostructures, with or without BDT. For the heterostructures, as one peak decayed, the peaks at later time scales were fit with only 2 Gaussians.

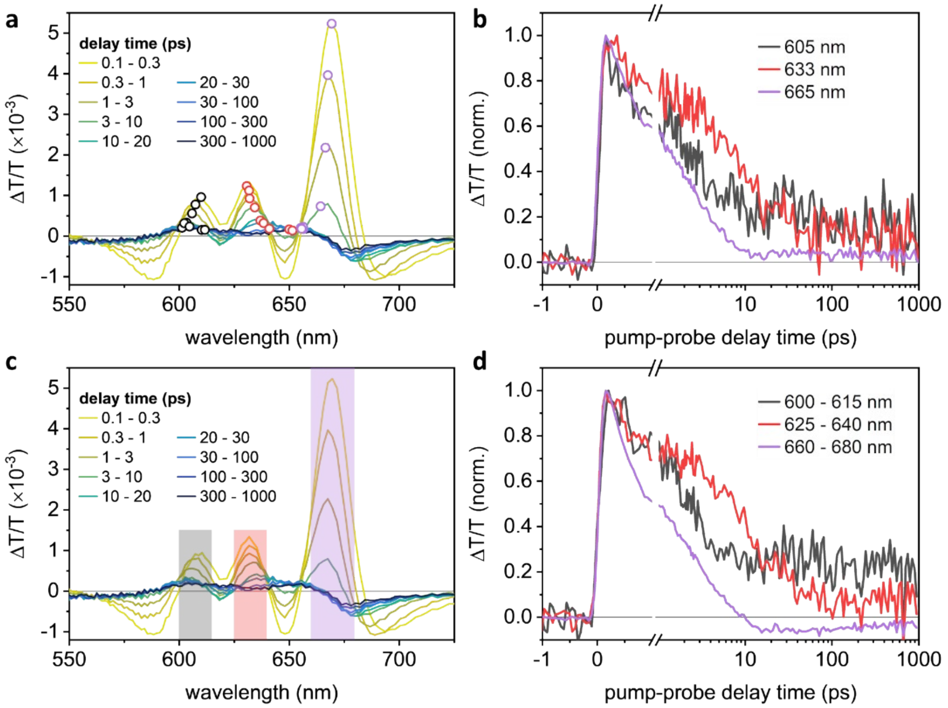

Figure S23. Example of tracing the kinetics of spectrally-shifting peaks (a,b) compared to tracing the kinetics by averaging over a fixed wavelength range (c,d). (a) TA spectra at different pump-probe delay times for pristine MoS_2-WS_2 heterostructure. Overlaid are the spectrally-shifting peak positions as determined from the multi-Gaussian fits. (b) Kinetic traces of the spectrally-shifting peaks as shown in (a) for each of the 3 dominant species: X_A of MoS_2 (~665 nm); X_A of WS_2 (~633 nm); and X_B of MoS_2 (~605 nm). (c) Same TA spectra from (a), but the overlay shows the fixed wavelength range where the kinetics were spectrally averaged. (d) Kinetic traces of the fixed wavelength ranges shown in (c). Note the X_A of MoS_2 signal drops to a negative value when the spectral shift is not taken into consideration.

8. Electrical characteristics in FET based on MoS_2-WS_2 heterostructure

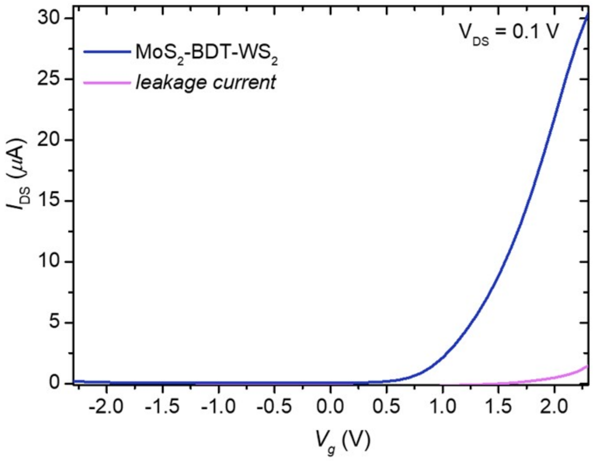

Figure S24. Leakage current in the champion device.

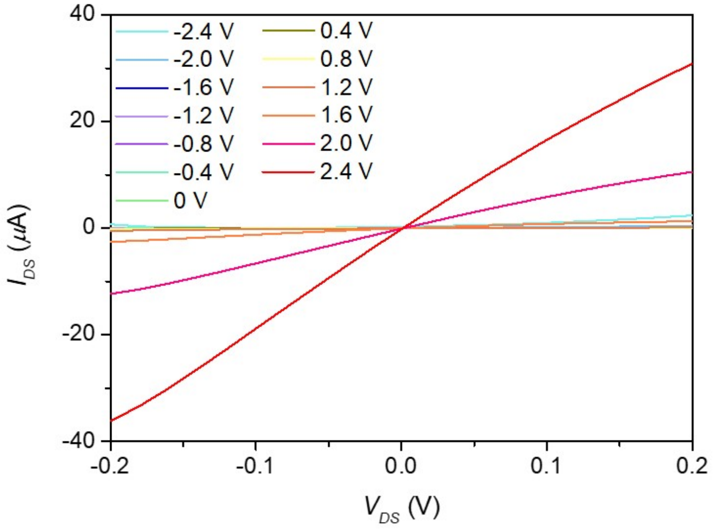

Figure S25. Output characteristics of an FET based on BDT-linked MoS_2-WS_2 heterostructure. Rectification behavior, typical of single MoS_2-WS_2 junction, cannot be observed in our BDT-linked MoS_2-WS_2 hetero-network because the drain current is averaged throughout the entire MoS_2-BDT-WS_2 channel network, as drain-source current must pass through multiple alternating WS_2-MoS_2 and MoS_2-WS_2 heterojunctions before reaching the source electrode. Also, it worth noting that both source and drain electrodes are in contact with different MoS_2 and WS_2 nanosheets simultaneously.

9. Field-effect mobility comparison

Film	μ_{FE} (cm^{-2} V^{-1} s^{-1})	I_{ON}/I_{OFF}	V_{th} (V)	SS (V dec^{-1})
MoS_2-BDT-MoS_2	(2.1 ± 0.2) x 10^{-2}	(1.0 ± 0.1) x 10^{2}	1.2 ± 0.2	0.63 ± 0.09
WS_2-BDT-WS_2	(7.6 ± 0.1) x 10^{-2}	(2.6 ± 1.3) x 10^{2}	1.7 ± 0.1	0.56 ± 0.02
MoS_2-BDT-WS_2	(1.6 ± 0.4) x 10^{-1}	(5.7 ± 2.0) x 10^{2}	1.5 ± 0.2	0.31± 0.16

μ_{FE} = field-effect mobility; I_{ON}/I_{OFF} = *ON:OFF ratio*; V_{th} = *threshold voltage; SS = subthreshold swing*

Table S3. Mean values and standard deviation of key parameters calculated from the FETs based on BDT networks with MoS_2, WS_2 and MoS_2/WS_2 building units. Measurements have been carried out by sweeping V_g between -2.3 and +2.3 V, with V_{DS} = 0.1 V.

Charge carrier mobility of the FETs was calculated using the following equation[1]:

$$\mu = \frac{L}{C_v \, V_{DS} \, W \, t} \frac{\partial I_{DS}}{\partial V_G} \quad (4)$$

where L_{ch} and W_{ch} are the channel length and width (10 μm each), respectively, V_{DS} is the source-drain voltage, t is the film thickness, C_v is the volumetric capacitance and dI_{ds}/dV_g is the maximum slope extracted from the linear region of the transfer curves. C_v values have been measured in our previous work,[1] which have been obtained by cyclic voltammetry in a three-electrode cell set-up with a Ag/AgCl reference electrode and a Pt counter electrode.

Film	Deposition	Thickness	μ_{FE} (cm^{-2} V^{-1} s^{-1})	Reference

		(nm)		
MoS_2-BDT-WS_2 network$^{(n)}$	microfluidic	45 ± 15	2.1 (1.6 ± 0.4) x 10^{-1}	*This work*
$MoS_2^{(n)}$	spray coating	500 ± 50	(1.4 ± 0.6) x 10^{-3}	*Adv. Mater.* **2023**, *35*, 221115
MoS_2-BDT network$^{(n)}$	spray coating	500 ± 50	(1.2 ± 0.7) x 10^{-2}	*Adv. Mater.* **2023**, *35*, 221115
$MoS_2^{(n)}$	ink-jet printing	20	(6 ± 2) x 10^{-2}	*Adv. Electron. Mater.* **2021**, *7*, 2100112
$MoS_2^{(n)}$	drop casting	700 ± 100	10^{-3}	*Nat. Nanotechnol.* **2021**, *16*, 592
$WS_2^{(n)}$	drop casting	700 ± 100	10^{-3}	*Nat. Nanotechnol.* **2021**, *16*, 592
$ReS_2^{(n)}$	drop casting	700 ± 100	10^{-4}	*Nat. Nanotechnol.* **2021**, *16*, 592
MoS_2-BDT network$^{(n)}$	drop casting	700 ± 100	10^{-2}	*Nat. Nanotechnol.* **2021**, *16*, 592
WS_2-BDT network$^{(n)}$	drop casting	700 ± 100	10^{-2}	*Nat. Nanotechnol.* **2021**, *16*, 592
ReS_2-BDT network$^{(n)}$	drop casting	700 ± 100	10^{-3}	*Nat. Nanotechnol.* **2021**, *16*, 592
$WS_2^{(n)}$	spray coating	150 ± 20	1.3 x 10^{-2}	*Adv. Funct. Mater.* **2018**, *29*, 1804387
$MoS_2^{(n)}$	spray coating	2600	1.5 x 10^{-1}	*Science* **2017**, *356*, 69
$WS_2^{(p)}$	spray coating	1800	2.2 x 10^{-1}	*Science* **2017**, *356*, 69

$^{(n)}$ = *n*-type; $^{(p)}$ = *p*-type

Table S4. Summary of μ_{FE} values based on various solution-processed 2D TMDs films. Mean values and standard deviation of μ_{FE} are in brackets, where available. It worth noting that TMDs films in cited works require thicknesses of hundreds/thousand nm to achieve sufficient percolation of charges.

References

1 A. G. Kelly, T. Hallam, C. Backes, A. Harvey, A. S. Esmaeily, I. Godwin, J. Coelho, V. Nicolosi, J. Lauth, A. Kulkarni, S. Kinge, L. D. A. Siebbeles, G. S. Duesberg and J. N. Coleman, *Science*, 2017, **356**, 69–73.
2 A. Syari'ati, S. Kumar, A. Zahid, A. A. E. Yumin, J. Ye and P. Rudolf, *Chem. Commun.*, 2019, **55**, 10384–10387.
3 C. Kastl, R. J. Koch, C. T. Chen, J. Eichhorn, S. Ulstrup, A. Bostwick, C. Jozwiak, T. R. Kuykendall, N. J. Borys, F. M. Toma, S. Aloni, A. Weber-Bargioni, E. Rotenberg and A. M. Schwartzberg, *ACS Nano*, 2019, **13**, 1284–1291.
4 B. Chakraborty, A. Bera, D. V. S. Muthu, S. Bhowmick, U. V. Waghmare and A. K. Sood, *Phys. Rev. B*, 2012, **85**, 161403.
5 E. P. Randviir and C. E. Banks, *Anal. Methods*, 2013, **5**, 1098–1115.
6 S. Wang, J. Zhang, O. Gharbi, V. Vivier, M. Gao and M. E. Orazem, *Nat. Rev. Methods Primers*, 2021, **1**, 1–21.
7 V. Montes-García, R. F. de Oliveira, Y. Wang, A. Berezin, P. Fanjul-Bolado, M. B. G. García, T. M. Hermans, D. Bonifazi, S. Casalini and P. Samorì, *Adv. Funct. Mater.*, 2021, **31**, 2008554.
8 M. Drüppel, T. Deilmann, P. Krüger and M. Rohlfing, *Nat. Commun.*, 2017, **8**, 2117.
9 P. Rivera, J. R. Schaibley, A. M. Jones, J. S. Ross, S. Wu, G. Aivazian, P. Klement, K. Seyler, G. Clark, N. J. Ghimire, J. Yan, D. G. Mandrus, W. Yao and X. Xu, *Nat. Commun.*, 2015, **6**, 6242.
10 A. Mondal, R. K. Yadav, M. Shrivastava, C. S. Rout, D. Karmakar and K. V. Adarsh, *Appl. Phys. Lett.*, 2020, **117**, 142104.
